# All‐in‐One Underwater Quality Evaluation Metamaterial With Mechanical Robustness, Sound Attenuation, and Diffuse Reflection

**DOI:** 10.1002/advs.202524261

**Published:** 2026-03-14

**Authors:** Hongze Li, Zhenyu Li, Jinshui Yang, Jianhao Wu, Yidan Chen, Linzhi Wu, Hong Hu, Penglin Gao, Yegao Qu

**Affiliations:** ^1^ College of Aerospace and Civil Engineering Harbin Engineering University Harbin P. R. China; ^2^ Qingdao Innovation and Development Base Harbin Engineering University Qingdao P. R. China; ^3^ School of Fashion and Textiles The Hong Kong Polytechnic University Hung Hom Hong Kong P. R. China; ^4^ School of Mechanical Engineering Shanghai Jiao Tong University Shanghai P. R. China

**Keywords:** fiber‐reinforced skeleton, heteromorphic structure, quality evaluation system, sound attenuation, underwater acoustic metamaterials, wave velocity reconstruction

## Abstract

To resolve acoustic‐mechanical conflicts and integrate research gaps in underwater coatings. Inspired by the biomechanics of jumping spiders and human bones, we design an underwater composite structure subject to hydrostatic pressure. Based on mechanisms involving weak energy entanglement driven by damping and wave‐mode conversion driven by impedance mismatch. A synergistic combination of theoretical modeling, numerical simulation, and experimental validation, the structure achieves low‐intensity diffuse reflection below 0.8 kHz, and broadband low‐frequency sound attenuation at 0.8–2.5 kHz (insulation > 26 dB, absorption > 0.8). Notably, this structure achieves significant sound attenuation with an absorption coefficient exceeding 0.8 below 4 kHz even under 3 MPa of hydrostatic pressure. The sound attenuation performance decreases by an average of only 4.5% per 1 MPa increase in pressure, and the deformation nearly 100% recovers after unloading. By integrating an acoustic‐electrical analogy model for component dimensionality reduction and a convolutional neural network for visual quality evaluation, we establish an integrated design‐evaluation framework. This strategy provides a scalable approach for next‐generation underwater acoustic skins.

## Graphical abstract

In this work, a bioinspired all‐in‐one underwater quality evaluation metamaterial, combining sound attenuation, diffuse reflection, and mechanical robustness, is proposed based on jumping spider locomotion and human skeletal biomechanics. Meanwhile, a CNN‑driven quality evaluation framework is established for theoretically dimension‐reduced heterogeneous units, facilitating performance assessment across an extensive parametric space.

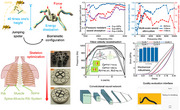

## 1 Introduction

Oceans and other water bodies cover approximately 71% of Earth's surface. Therefore, underwater acoustics is a vital research area, spanning sound detection, stealth [1, 2], and manipulation [3]. For sound stealth technology, effective absorption and diffuse reflection of low‐frequency broadband sound waves are critical. However, this field remains less mature than air‐mediated vibration and noise control [4] or ultrasound imaging [5, 6]. For airborne noise reduction, the primary advantages arise from the energy storage and dissipation properties [7] of the composite's local triboelectric effect [8] and the local resonance property [9] of acoustic metamaterials. However, the air‐water impedance difference [10] compromises the low‐frequency sound attenuation advantage of acoustic metamaterials [11]. It raises questions about the applicability of the local resonance mechanism for low‐frequency attenuation [12]. This limitation occurs because dissipating large‐wavelength sound waves in water requires a larger material impedance mismatch [13] to drive wave‐mode conversion [14, 15]. Consequently, underwater sound attenuation research has focused on structural optimization to enhance wave‐mode conversion efficiency [16, 17, 18, 19, 20]. However, efficient sound wave coupling into the surface layer material is a prerequisite for this process, while almost all existing work neglects to distinguish between surface materials and matrix materials [14, 21, 22, 23, 24].

Furthermore, the sound attenuation properties of metamaterials are dramatically reduced under hydrostatic pressure [22, 23, 24], and the honeycomb‐reinforced configuration is often employed to maintain acoustic performance [25]. However, honeycomb wall thickness limits the size of the metamaterial single unit. Although honeycomb‐reinforced configurations exhibit high specific strength and stiffness [26], their pressure adaptability remains inadequate for complex underwater hydrostatic variations. Therefore, structural mechanical robustness requires improvement. Notably, similar challenges have emerged in airborne noise reduction, where existing solutions may inform mitigation strategies. Acoustic‐mechanical multifunctional structures integrate acoustic metamaterials and deformation‐resistant or deformation‐recoverable structures through coupled design [27, 28, 29, 30, 31]. This approach enhances mechanical properties while preserving sound attenuation capabilities. Specifically, lattice and auxetic structures [32, 33] exhibit superior deformation compatibility compared to honeycomb configurations [34]. Meanwhile, their acoustic design flexibility, particularly the ability to shift and broaden target frequency bands, offers greater optimization potential than traditional Helmholtz resonator series‑parallel configurations. The aforementioned multifunctional structures exhibit multi‑objective performance enhancement capabilities [35]. These capabilities significantly broaden their application potential beyond that of conventional acoustic and mechanical metamaterials. However, a research gap persists in achieving underwater pressure adaptation for these structures while preserving their acoustic performances. Unlike airborne noise solutions (e.g., membrane‐type metamaterials [36] or Helmholtz resonators [37, 38]), underwater sound attenuation prioritizes the integration of impedance‐mismatched materials. Concurrently, designing mechanically integrated components remains a critical challenge.

The preceding discussion focuses on pressure‐resistant metamaterials for the attenuation of underwater sound. However, most current studies target a single objective (e.g., sound attenuation), neglecting the multi‐objective optimization required for effective sound stealth. On the other hand, the acoustic impedance gradient between pressure‐resistant absorbers and water, as well as wave velocity anisotropy, governs sound scattering intensity [2]. Engineering structures can enhance acoustic concealment by reducing the intensity of sound echoes. However, impedance modulation exhibits a pronounced antagonism between sound absorption, scattering, and load bearing. Optimizing this compromise remains a significant challenge.

In our previous work, we have investigated the local resonance mechanism and impedance‐matching properties of acoustic metamaterials [39]. Simultaneously, we construct the performance‐geometry‐structure mapping using a deep neural network, enabling the inverse design of the structure [40]. Additionally, we integrate acoustic and mechanical metamaterials through coupled design to optimize their joint acoustic‐mechanical performance [30]. Here, based on the above theoretical framework, inspired by the biomechanics of Salticidae jumping and human skeletal systems, we have designed an underwater acoustic composite structure that combines mechanical robustness with sound stealth. Concurrently, we develop a metamaterial quality evaluation interface using optimization theory and convolutional neural network‐based image recognition [41] to visualize structure‐function relationships. In what follows, we decompose the structure into parallel subunits. Inspired by hemolymph viscous dissipation in salamander jumping, we enhance the damping of heteromorphic structures to improve acoustic energy dissipation. Simultaneously, vertebral‐rib‐inspired mechanical skeletons enable pressure adaptation at larger scales. We characterize performance from multi‐scale material‐structural perspectives and formulate the reconfigured system's sound stealth using logic gates. Ultimately, the realized structure achieves low‐frequency sound diffuse reflection and full‐frequency attenuation while integrating mechanics and intelligent control for underwater acoustic skins.

## 2 Results

### 2.1 Multi‐Functional Coupled Design Process and Evaluation System

Acoustic metamaterials (AMs) demonstrate fundamentally distinct material and structural behaviors in aqueous environments compared to atmospheric conditions, particularly when engineered at subwavelength scales to simultaneously address hydrostatic pressure challenges and achieve efficient low‐frequency sound attenuation. This cross‐disciplinary challenge stemming from hydrostatic loading and multi‐environment adaptability necessitates a synergistic design framework that co‐optimizes metamaterial architecture across four critical dimensions: structural geometry, material composition, functional response, and quality evaluation. Such an integrated approach has successfully established a non‐linear design paradigm that achieves unprecedented synergy between acoustic functionality (sound attenuation and diffuse reflection) and structural integrity (pressure resilience and robustness), effectively overcoming the historical dichotomy in underwater metamaterial development. This metamaterial architecture demonstrates functionally stratified design principles through dual‐physics mechanisms: local resonance modulation and broadband impedance matching. The tripartite system comprises (i) a multifunctional matrix governing sound wave absorption and structural load‐bearing (acoustic impedance gradients and stress redistribution), (ii) a parallel skeleton implementing energy dissipation and hydrostatic adaptation (pressure‐adaptive topologies), and (iii) resonant scatterers enabling multifunctional synergies (thermo‐viscous dissipation and coupled vibrations). Such categorization achieves spatiotemporal coordination of wave manipulation and mechanical performance, establishing a new design paradigm that transcends conventional monofunctional metamaterial limitations.

**FIGURE 1:**
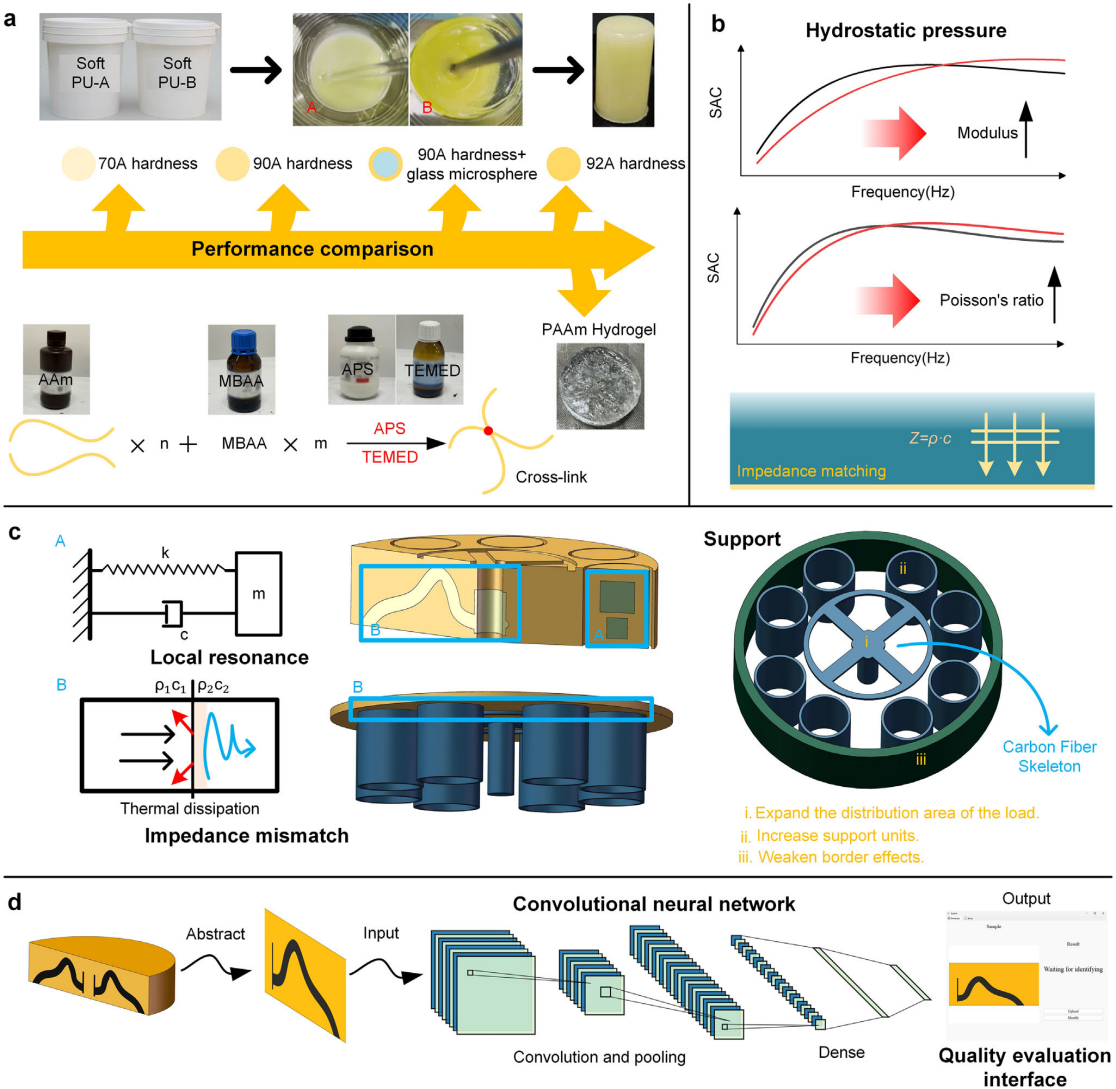
Underwater quality evaluation metamaterial: coupled design framework and quality decision interface. (a) Comparison of matrix material properties and selection of functionalization. It mainly contains PU and hydrogel, taking into account sound attenuation, sound diffuse reflection (Movie S1), and mechanical robustness. (b) The case of the influence of possible material parameters under hydrostatic pressure, with the main focus on modulus and Poisson's ratio. (c) A coupled acoustic‐support unit design based on local resonance and impedance matching mechanisms, in which the support adopts a fiber skeleton to extend the load distribution area and weaken the boundary effect. (d) The acoustic units are abstracted in 2D, several images are generated for CNN training, and the labels are defined to set the quality evaluation interface.

The material selection framework employs a dual‐phase systematic investigation, strategically contrasting synthetic polymers with biomimetic hydrogels to establish universal design principles. For polyurethane (PU)‐based matrices, we have developed a multi‐parameter screening platform that decouples acoustic‐mechanical interdependencies through controlled Shore hardness gradients (70–92 A) and precisely added hollow glass microspheres (9 vol%), as shown in Figure 1a. This novel composite material enables concurrent parametric dependencies in sound absorption coefficients (0.2–10 kHz band), compressive modulus (10–40 MPa range), and Poisson's ratio (>0.45), revealing monotonic property relationships governed by material parametric trends under hydrostatic pressure conditions (Figure 1b). Complementarily, the hydrogel implementation constitutes a bio‐inspired material strategy achieving 90% acoustic impedance transparency (Z = 1.67 MRayl vs water's 1.5 MRayl) through programmable polymer network crosslinking density (0.1 mol/L N, N‐methylenebisacrylamide: MBAA). It is hypothesized that coating the upper surface of structural components with hydrogel matrices can significantly improve acoustic energy transduction efficiency by enabling directional wavefront propagation and targeted energy dissipation within the internal architecture (Figure S3f).

Employing a bio‐inspired design framework derived from spider hydraulic actuation mechanisms and human musculoskeletal biomechanical systems, we have developed macroscale engineered composites with a multiparametric acoustic‐mechanical structure. This approach synergizes local resonance phenomena with impedance gradient mismatch, contrasting with conventional underwater metamaterials reliant on homogeneous honeycomb topologies. Figure 1c demonstrates that pronounced impedance contrast within the composite structure induces wave‐mode conversion, driving thermal dissipation through which mechanical energy is transduced into thermal and effectively dissipated via interfacial friction mechanisms. On the other hand, building on air‐media AMs, we propose a targeted local resonance strategy to enhance subwavelength‐scale low‐frequency attenuation through mechanical energy storage and frequency‐selective dissipation mechanisms. We have equated the scatterer and matrix to a resonant spring‐mass interface, achieving controlled natural frequency shifting into the low‐frequency regime through targeted vibrational mode coupling. Spectral matching between incident waves and resonator subsystems induces resonant amplification, significantly amplifying acoustic energy dissipation through targeted mechanical energy conversion pathways. In addition, we have engineered a pressure‐resistant composite structure through the coordinated preparation of macro‐architected fiber‐reinforced skeletons and viscoelastic polymer matrices, thereby abandoning conventional honeycomb geometries to achieve simultaneous enhancement of hydrostatic stability and mechanical robustness.

This study elucidates the geometry‐function interplay in spider‐like metamaterial architectures for underwater quality evaluation, demonstrating geometry‐dependent sound attenuation capabilities through biomimetic design principles. Bioinspired underwater composite structures integrating arachnid‐inspired elastic inclusions within a PU matrix achieve radial mechanical energy dissipation through an impedance gradient mismatch, demonstrating enhanced low‐frequency sound attenuation (0.2–2 kHz) via wave‐mode conversion mechanisms and multiscale interfacial damping. Equivalent circuit analysis via acoustic‐electrical analogy reveals the bioinspired composite system‐ comprising a spider‐like elastomeric core and PU matrix‐ as an impedance‐parallel topology, enabling decomposition into discrete energy‐dissipative pathways through parallel resistors equivalent mechanisms. The rotational symmetry (C_4_) inherent to the 90° equiangular elastomeric architecture enables uniform radial energy dissipation profiles, which can realize the two‐dimensional (2D) abstraction assumption. Leveraging this validated theoretical framework (Figure S1), we establish a numerical computation‐data augmentation passage that generates 2D image datasets for training a convolutional neural network (CNN), enabling the mapping model to be obtained through machine learning‐derived geometry‐functional correlations [42]. Next, we have developed a machine learning‐compatible evaluation framework for underwater metamaterial systems, integrating quantitative performance metrics (sound absorption coefficients) with a visualization platform (Figure 1d), reflecting the sound attenuation quality of the composite structure.

### 2.2 Design Philosophy and Analytical Strategies of Spider‐Like Heteromorphic Structure

**FIGURE 2:**
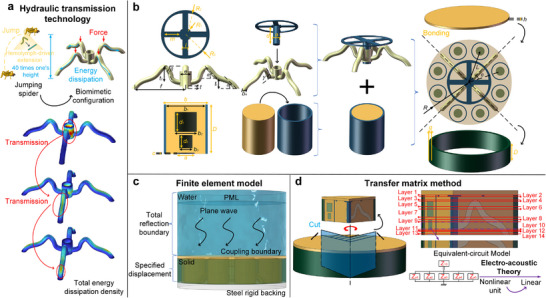
Integrative bioinspired acoustic‐design framework. (a) An energy dissipation model inspired by the hydraulic transmission technology of Salticidae. (b) Structural components and related dimensions. (c) Multiphysics field discrete numerical model for acoustic analysis. Including solid‐liquid two‐phase coupling relationship, confinement effects, dynamic excitation modalities, etc. (d) Conventional TMM augmented through electroacoustic analogies with 50% predictive accuracy enhancement.

Jumping spiders (Salticidae), a widespread group of arachnids within the arthropod phylum, exhibit extraordinary leaping capabilities through specialized hydraulic limb extension mechanisms, with biomechanical analyses documenting vertical jumps exceeding 40 times their body length. This biomechanical feat in salticids is achieved through rapid abdominal muscle contractions that pump body fluids into the legs at extreme speeds‐ a mechanism termed the hydraulic transmission technique in arthropod locomotor studies. Drawing on the thermodynamically coupled biomechanics of Salticidae locomotion‐ where viscous dissipation of hemolymph during rapid pressurization induces transient temperature elevation‐ we have engineered a spider‐like heteromorphic structure, as shown in Figure 2a. Incident wave propagation induces multimodal vibrational energy transduction across media interfaces, where kinetic energy partitions between dissipative thermal conversion through non‐conservative interfacial interactions and conservative storage as elastic potential energy within materials. This metastable equilibrium undergoes rapid hierarchical energy redistribution, where stored potential energy is partially converted into kinetic energy while residual energy dissipates through inherent viscoelastic relaxation pathways governed by molecular‐scale anharmonic interactions. This energy dissipation dynamic system achieves sound attenuation through impedance‐mediated acoustic energy‐mechanical energy transmission followed by intermedium dynamics‐driven thermalization, establishing a self‐sustained energy transduction continuum governed by non‐equilibrium thermodynamics.

Figure 2b illustrates the structural preparation logic and the dimensions of each component. The specific geometric parameters are shown in Table S1. In this study, the sound attenuation performance of the structure is investigated through a combination of theory, simulation, and experiment, as shown in Figure 2c,d. Specifically, a multiphysics field‐coupling strategy is implemented for structural discretization analysis, with subsequent sound absorption coefficients of the composite structure being systematically determined through the transfer matrix method (TMM). Notably, whereas the conventional TMM remains predominantly applicable to linearly arranged elementary unit cells, it demonstrates constrained efficacy in characterizing the multiscale nonlinear architectures developed herein. To overcome this limitation, we integrate electroacoustic analogy with the TMM to establish structural equivalence. This framework decomposes the developed multiscale nonlinear architectures into discrete linear subunits, enhancing computational precision by ≈50% (Δe vs. conventional methods).

### 2.3 Matrix Pressure Adaptation and Microscopic Analogy

**FIGURE 3:**
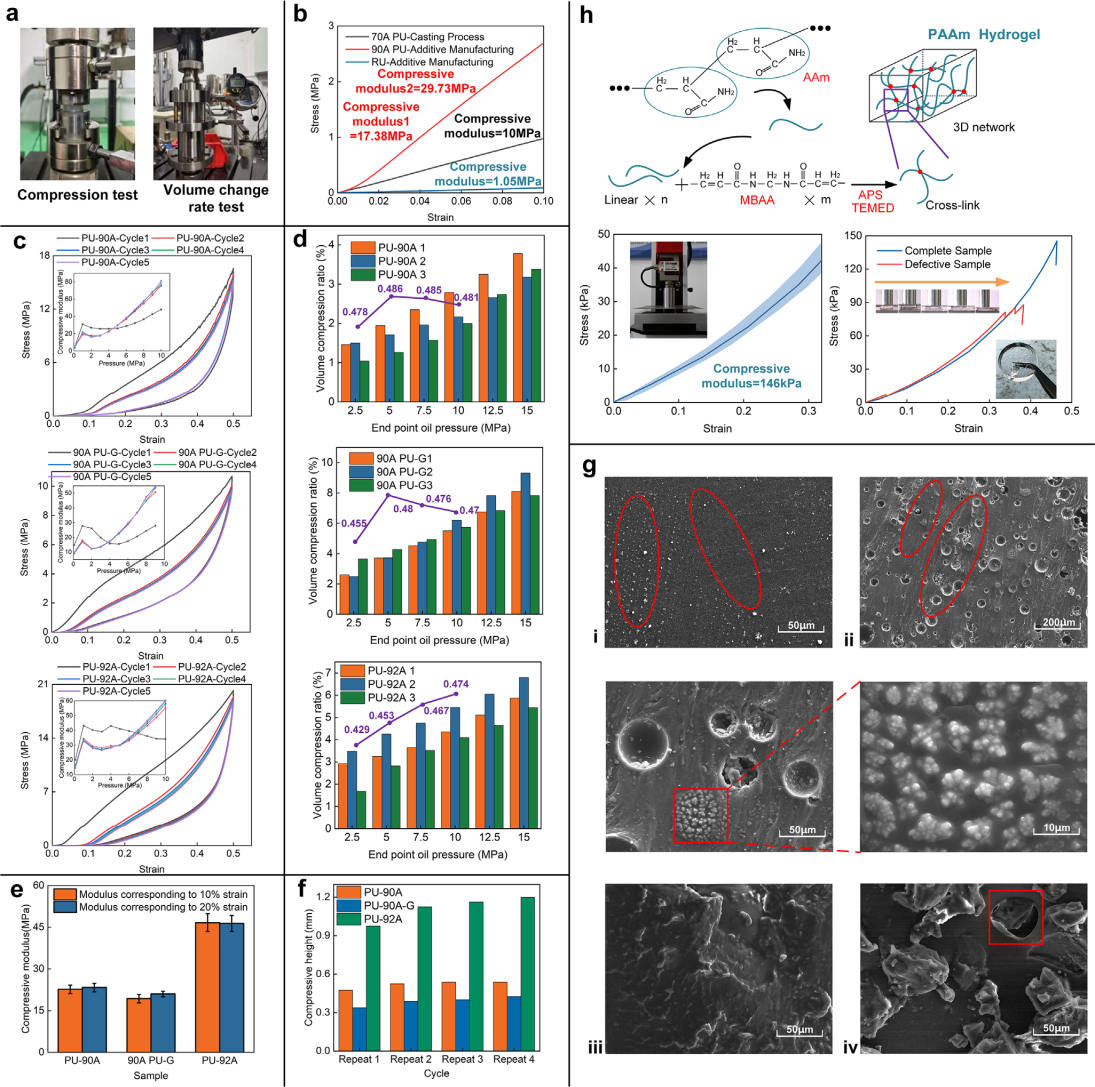
Pressure adaptivity and microscopic analogs of candidate matrix materials. (a) Electro‐mechanical universal testing machines and hydraulic testing systems for mechanical property characterization and volumetric change rate measurements. (b) Stress‐strain curves of PU (Shore 70A and 90A) and rubber (RU). (c) Stress‐strain curves of three matrix materials under 5‐cycle loading conditions: mixed PU (Shore 90A), PU/glass microsphere (K25) composite (Shore 90A), and 3D‐printed PU (Shore 92A). Meanwhile, a quantitative analysis has been performed to characterize the variation in material modulus as a function of increasing stress. (d) Volumetric compression radios of three matrix materials under 2.5–15 MPa compressive loading: 90A PU, 90A PU/glass microsphere composite, and 92A PU. (e) Compressive modulus of three materials under 10% and 20% strain. (f) Deformation recovery characteristics of materials under cyclic loading. (g) The microscopic analogs of three materials: Soft/Hard segment and glass microsphere distribution (i‐ii), particle size (ii), process differences (iii), and damage (iv). (h) Network architecture characterization and compressive stress‐strain behavior of PAAm hydrogels under controlled loading regimes.

This section systematically evaluates the performance metrics of the multi‐parameter matrix material screening platform (Figure 1a), focusing on its capability to resolve critical structure‐property relationships under combinatorial testing conditions. Our analysis prioritizes the acoustic performance of the system under 0.1 MPa hydrostatic pressure (equivalent to 10‐meter water depth), where strains remain nearly 1%, thus justifying the application of a linear elastic constitutive model for acoustic‐solid coupling analysis. Using electro‐mechanical universal testing machines and hydraulic testing systems (Figure 3a), we have conducted a systematic analysis of modulus and Poisson's ratio across multiple stress regimes (Figure 3b,c,d,e), demonstrating statistically validated evolution of the modulus and Poisson's ratio of the materials. Meanwhile, the deformation recovery property of PU‐based materials enables pressure‐adaptive functionality (Figure 3f). The microscopic analogy of soft/hard segment and filler dispersion homogeneity, as well as interfacial integrity, has revealed strong correlations between processing parameters and mechanical performance (Figure 3g). Furthermore, to address the dual‐phase performance requirements of optimized acoustic impedance matching and effective sound attenuation‐ essential for enabling unimpeded sound wave penetration through the structural interface followed by internal energy dissipation‐ we propose a material selection strategy prioritizing substances with superior acoustic impedance matching with aqueous environments. Hydrogels emerge as the candidate material for the surface layer due to their unique combination of water‐like acoustic properties (ρc ≈ 1.5 MRayl) and tunable viscoelastic characteristics. These facilitate efficient sound wave transmission through the structural surface for subsequent energy dissipation within the internal components. Systematic comparisons of complete and defective hydrogel specimens reveal distinct mechanical behaviors, with a significant reduction in mechanical stability caused by structural defects. Meanwhile, the statistical analysis of the tests has confirmed that a 14 wt% polyacrylamide (PAAm) hydrogel approximately exhibits an elastic modulus of 146 kPa (Figure 3h).

In contrast to air media, the underwater acoustic characterization of metamaterial systems reveals that sound propagation phenomena become critical determinants of performance, intricately dictated by constituent material properties and structural composition. Incorporating viscoelastic materials with elevated sound transmission coefficients and favorable molecular thermal dynamics as matrix components represents a critical prerequisite for ensuring effective underwater sound attenuation. This study employs PU and hydrogel as representative material systems to investigate pressure adaptive performance, establishing a comparative framework to analogize parametric change trends of key material characteristics under varying hydrostatic conditions. The experimental methodology employs a segmented analytical framework, wherein multistage compression analysis of mixed PUs (Shore 70 A, 90 A) enables mechanistic decoupling through Figure 3b–e. These distinct deformation regimes correspond to: (1) the linear elastic regime (*ε* < 1%) demonstrating minimal hydrostatic coupling effects, and (2) the nonlinear viscoelastic regime exhibiting pressure‐modulated modulus evolution. Experimental characterization (Figure 3b) reveals distinct compressive responses between material systems: 70A PU composites exhibit a compressive modulus near 10 MPa (8.85–10 MPa), while 90A formulations approximate 20 MPa (17–30 MPa). Based on the results in Figure 3c, we have analyzed the deformation under cyclic loading for three materials: 90A PU, its composite with glass microspheres, and 92A PU fabricated via additive manufacturing. Under simulated near‐water‐surface conditions (stress near 0.1 MPa, strain near 0.01), the PU matrix composite exhibits linear elastic behavior. During this stage, the load is predominantly borne by the soft‐segment phase, resulting in a relatively low modulus‐ approximately 15%–50% of the conventional hardness value for the same material. To simplify the analysis and decouple the nonlinear deformation of the viscoelastic polymer, we propose a hypothesis inspired by the definite integral method: the macroscopic nonlinear region can be described as a series of small‐range linear deformation segments. This approach allows us to derive the variation in modulus under large deformation, shown in Figure 3c. In contrast, during the initial nonlinear stage (stress between 0.1 and 1 MPa), stress becomes concentrated in the hard‐segment phase, and the primary load‐bearing role shifts from the soft‐segment phase to the hard‐segment phase, leading to a sharp increase in the modulus. The above analysis identifies two governing parameters in PU compression mechanics: (1) intrinsic viscoelastic compressibility (characterized by pressure‐dependent compressive modulus and Poisson's ratio) and (2) deformation recovery after unloading and reusability. This multiscale framework quantitatively reconciles macroscopic modulus variation with microstructural evolution mechanisms.

The volumetric compression behavior of a PU elastomer (Shore 90A) is systematically investigated under different pressure conditions ranging from 0 to 15 MPa (increase gradient 2.5 MPa). The pressure‐dependent Poisson's ratio ν is quantitatively determined through computational analysis using Equation (1).

(1) $$\frac{\Delta V}{V}=-\frac{P\cdot 3\left(1-2\nu \right)}{E}$$

where the volume compression Δ*V* can be obtained by experiment according to the corresponding pressure *P*. These dimensional parameters enable precise calculation of sample volume (*V*) through geometric determination using the formula for cylindrical volume (*V* = π*r* ^2^ *h*), where *r* represents radius (14.75 mm) and *h* denotes height (12.5 mm).

Quantitative analysis of Figure 3c,e shows a statistically significant rise in the compressive modulus of the material at stresses between 0.1 and 1 MPa. Subsequently, under linearly approximated conditions, the modulus decreases slightly at higher stresses before stabilizing and increasing steadily. This trend reflects the stabilized mechanical response of the mixed system when force‐evolving microstructural rearrangements are computationally decoupled from bulk deformation behavior. In contrast, traditional strain‐driven approaches typically assess modulus variations within the 10%–20% strain range, where the compressive modulus shows only a minor increase (ΔE = 0.67 MPa, corresponding to a 2.87% enhancement). This method, however, overlooks multiple characteristic deformation stages and may therefore lead to misinterpretations of material behavior. Furthermore, the volumetric analysis within the 2.5–15 MPa pressure regime has demonstrated a mean compressive change rate of 2.12%, with Equation (1)‐derived Poisson's ratio evolution exhibiting asymptotic stabilization, as numerically documented in Figure 3d.

The described mechanical behavior can be explained through macroscopic analysis at each deformation stage. Under initial stresses ranging from 0 to 2.5 MPa, the modulus of 90A PU gradually increases to a maximum before declining or plateauing, while exhibiting a low Poisson's ratio (maximum 0.478). This allows the material to undergo substantial volumetric compression without losing structural integrity. During this stage, the material exhibits a greater tendency toward compression‐induced contraction in the direction of applied pressure, accompanied by delayed vertical expansion. Such unique compressive behavior enhances its displacement response upon initial exposure to external loads. Under progressive mechanical loading, PU transitions into an advanced deformation regime characterized by the dynamic evolution of its modulus and Poisson's ratio. The modulus of the material increases gradually, while its Poisson's ratio progressively converges toward a stable value. This trend reflects a transition to a steady mechanical state, accompanied by a reduction in volumetric compliance. These internal adjustments manifest primarily as enhanced compressive resistance along the stress direction and reduced expansion hysteresis perpendicular to it. Together, these changes drive an increase in the material's bulk modulus. Microstructural analysis (Figure 3g‐i) clarifies the origin of the sharp rise in the PU modulus observed between 0.1 and 1 MPa. The material contains polydisperse micron‐sized hard segment phases whose spatial distribution relative to surrounding soft segments creates distinct stress pathways. Since the hard‐segment modulus significantly exceeds that of the soft segments, initial low‐stress deformation is accommodated by soft‐segment chain motion, yielding a lower overall modulus. With increasing stress, load transfer concentrates on the hard segments, which begin to bear the load predominantly, resulting in the observed step‐like increase in modulus. Simultaneously, the load‐bearing capacity of the hard segments restricts the lateral stretching of the soft‐segment network perpendicular to the compression direction. This reduction in expansion decreases the available free volume, which in turn suppresses the increase in Poisson's ratio.

Subsequently, 9 vol% hollow glass microspheres (K25, nominal strength 5.17 MPa) are incorporated into the mixed PU matrix (Shore 90A) through controlled mechanical mixing. The composite specimens are subjected to compression testing to determine their elastic modulus and volumetric compression characteristics, with corresponding results presented in Figure 3c,d. Research demonstrates that incorporating hollow glass microspheres into PU markedly enhances its compressive modulus. At 0.1 MPa stress, the modulus rises to 313% of that of the pure material (from 4.72 to 14.78 MPa). However, as stress increases to 1 MPa, the modulus declines to 90% of the raw PU value (from 30.78 to 27.75 MPa). Under stresses above 1 MPa, the modulus shows an average reduction of approximately 30% compared with the unmodified material. Additionally, the inclusion of glass microspheres increases the volumetric compression ratio of PU by a factor of 2.5. Macroscopic compression testing reveals that the reinforcing effect of glass microspheres in PU is most pronounced at low stress levels (0.1 MPa). Concurrently, the calculated Poisson's ratio shows a wider range of variation than that of neat PU, decreasing notably at high stress (from 0.48 to 0.47). These results indicate that the incorporation of hollow glass microspheres directly enhances the compressibility of the composite, thereby reducing the effective bulk modulus of the material system. In contrast to PU‐ a nearly incompressible elastomer‐ and high‐strength brittle glass, hollow glass microspheres containing encapsulated air exhibit significantly compromised mechanical strength. From a compositional perspective, incorporating glass microspheres reduces the PU content in the composite system, thereby decreasing Poisson's ratio. Microscopic analysis of Figure 3g‐ii reveals a random dispersion of hollow glass microspheres within the PU matrix, characterized by stochastic variations in spatial distribution (∼200 µm spacing) and particle diameters (∼50 µm spacing). At the low stress level (0.1 MPa), glass microspheres act in place of the hard segments to help support the load within the soft segments, leading to a marked rise in compressive modulus. As stress increases, particle agglomeration across size variants (∼10–50 µm spacing) induces microscale stress concentrations, triggering premature fracture of glass microspheres at critical stress thresholds. This fracture cascade propagates interfacial instability and amplifies structural heterogeneity within the composite. The degradation phenomenon indirectly diminishes the compressive modulus, Poisson's ratio, and other parameters that characterize mechanical properties. Furthermore, environmental exposure to sunlight, temperature, and humidity induces oxidative and hydrolytic degradation in the mixed PU, leading to gradual hardening and a consequent loss of its initial viscoelastic properties over time. The above phenomenon is a critical consideration for applications requiring mechanical stability. To address these limitations, we implemented precision additive manufacturing for PU processing, selecting Shore 92A‐grade material (supposed as the curing limit of Shore 90A specimens) for multiscale mechanical evaluation spanning macroscopic mechanical testing to microscale characterization. Quasi‐static compression tests show that the PU modulus approximately doubles over the stress range of 0.1–1 MPa, and then remains largely constant at higher stresses. Concurrently, its Poisson's ratio increases progressively, asymptotically approaching a limiting value of 0.475. Figure 3g‐iii shows that precision additive manufacturing PUs exhibit enhanced continuity and stable mechanical performance compared to conventional counterparts. Under extreme loading, micropore formation occurs in the material (Figure 3g‐iv), attributed to incomplete elastic recovery from localized molecular chain rupture or slippage. However, minimal gas entrapment negligibly compromises the material's reusability.

PU‐based composites demonstrate both high compressive strength and resilient deformation recovery, enabling repeated reuse. In cyclic loading tests (second to fifth cycles, Figure 3c,f), the compressive modulus exhibits a tiered phenomenon (approximately 4.5 MPa for 90A PU, 3.9 MPa for the PU/glass microsphere composite, and 6.8 MPa for 92A PU). Relative to the first cycle, the modulus decreases at lower stresses but increases at higher stresses, with subsequent cycles showing no significant further variation. This behavior indicates stable mechanical performance under repeated large‐strain (50%) loading. Moreover, all three materials retain over 90% of their instantaneous rebound after five cycles. Any minor residual deformation recovers nearly completely within 24 h through delayed elastic recovery, ensuring reliable pressure adaptability.

Under hydrostatic pressure‐negligible near‐surface aquatic environments where water temperature closely tracks ambient air temperature, and humidity remains near saturation, employing hydrogel surface layers with optimized acoustic impedance matching enables near‐perfect sound wave transmission through the water‐material interface [43]. The hydrogel synthesis protocol (Figure 1a), corresponding molecular cross‐linking mechanism [44], and characterization of mechanical properties (Figure 3h) demonstrate that 14 wt% PAAm hydrogels exhibit a compressive modulus of 146 kPa under loading‐ approximately two orders of magnitude lower than Shore 70A PU (Figure 3b). Meanwhile, the material exhibits brittle failure at 46% strain (Figure 3h), characterized by catastrophic fragmentation and complete loss of stability under uniaxial compression. This reveals its inherent inability to dynamically reconfigure (modulus, Poisson's ratio) for hydrostatic pressure adaptation. Boundary defects (5% notch introduced at hydrogel edges) have significantly reduced compressive strength by 44% (145.29–81.32 kPa) and ultimate load‐bearing strain from 46% to 38%, demonstrating defect‐driven mechanical destabilization under pressure [44, 45]. The failure process has exhibited progressive delamination initiating from boundary defects, with two distinct interlayer fractures observed at 34% and 36% strain thresholds, highlighting structural vulnerability to stress localization. In summary, hydrogels exhibit superior acoustic impedance matching with aqueous environments and high structural designability. However, it exhibits critical limitations in hydrostatic pressure adaptability and environmental sensitivity to temperature/humidity [46]. Nevertheless, hydrogels are still promising candidates for near‐surface acoustic structures. Their suitability arises from excellent sound attenuation, a result of high impedance matching with water (Figure S3).

### 2.4 Mechanical Robustness Enhancement Realized by Muscle‐Skeleton Analogy

Designing pressure‐resistant acoustic skins for the streamlined surface of underwater vehicles requires addressing multiscale structural adaptability in composite systems. Honeycomb structures emerge as the optimal configuration in the previous study, balancing mechanical stability with sound attenuation performance [31]. However, conventional honeycomb structures face inherent acoustic scaling constraints. Their load‐bearing capacity and sound absorption exhibit competing demands governed by cell‐wall thickness ratios, ultimately limiting applications in large‐scale streamlined systems [47]. Furthermore, scale constraints further limit honeycomb structural design flexibility.

**FIGURE 4:**
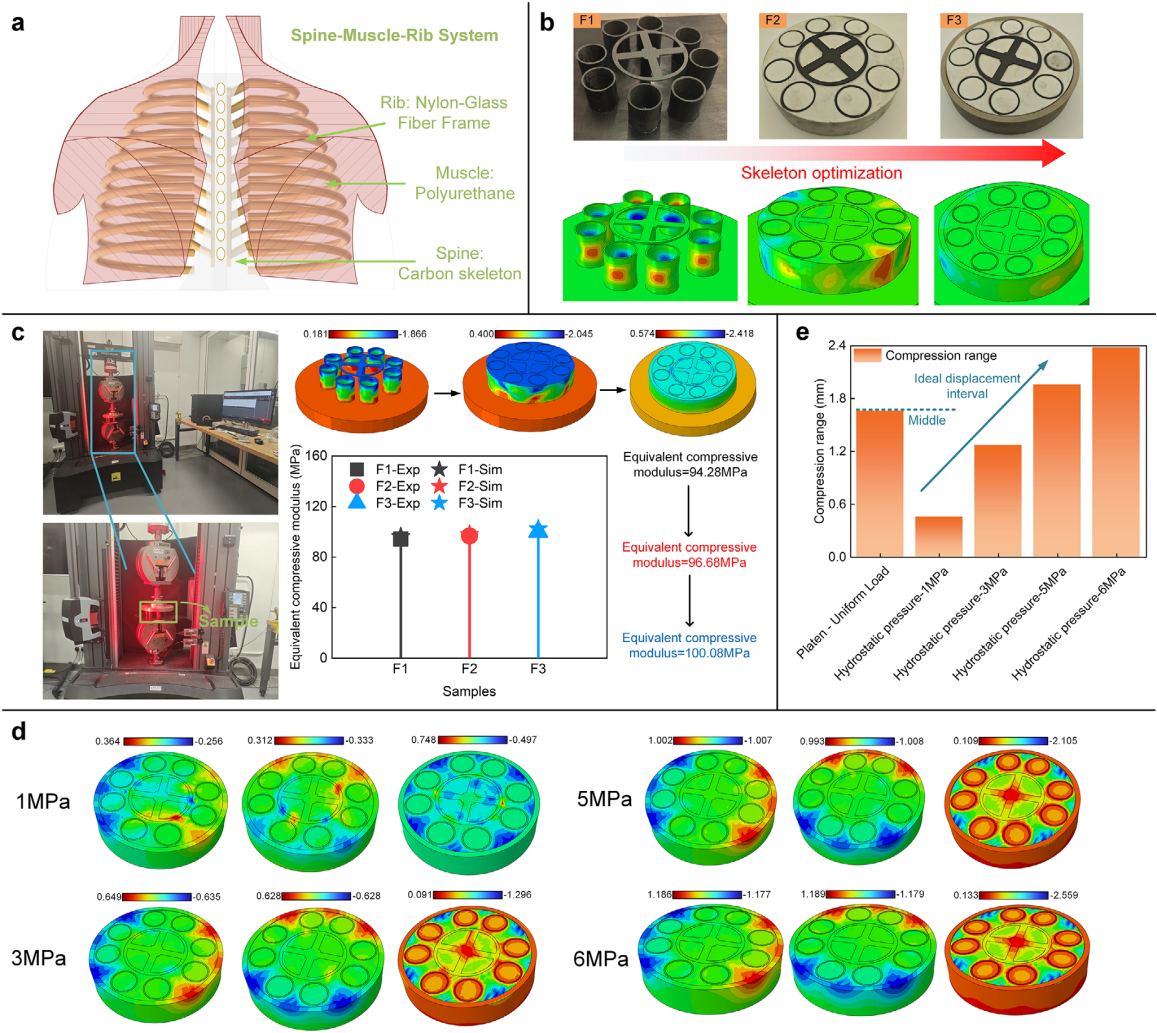
Biomimetic hydrostatic strategy in musculoskeletal architectures‐ enhancing mechanical robustness in underwater systems. (a) Triphasic bioinspired architecture‐ fiber reinforced polymer (FRP) ‐PU hybrids for multiple load adaptation. (b) Structural configurations for skeleton reinforcement. (c) Experimental characterization of quasi‐static compression. (d) Compressive analogy for different hydrostatic conditions. (e) Analogical feasibility analysis.

The heteromorphic mechanical skeleton overcomes conventional honeycomb constraints through programmable geometry. By integrating elastomeric components, this design simultaneously enhances mechanical robustness and acoustic damping, resolving the persistent acoustic‐mechanical compromise in underwater surface engineering. As depicted in Figure 4a, the human body integrates two hierarchical tissue phases‐ structural and mechanoadaptive‐ that synergistically sustain biomechanical integrity during homeostatic regulation and high‐intensity physiological exertion. The organism achieves environmental adaptation through neuromuscular modulation of skeletal kinematics. This process involves myofascial actuation generating compressive forces to drive dynamic postural reconfigurations. Furthermore, this work hierarchically integrates acoustic‐mechanical systems by mimicking the above content. Metamaterial skins engineered for underwater vehicles must adapt to dynamic variations in hydrostatic pressure across different diving depths (Figure S7). This pressure‐responsive behavior parallels biological homeostasis in humans, where mechanotransduction pathways enable physiological adaptation to environmental stresses. We have engineered a load‐bearing skeleton using fiber‐reinforced composites, which synergistically integrate low density, exceptional mechanical strength, and sound transmission capabilities. The vertebral‐inspired endoskeletal architecture and ribcage‐mimetic exoskeletal counterpart have been engineered with fiber‐reinforced composites as the primary structural materials, interconnected by the pressure‐responsive PU matrix to enable dynamic pressure adaptation (Movie S2) and acoustic energy dissipation. The enhanced structure comprises three functionally graded components: (1) a carbon fiber‐reinforced composite (T300 fabric) vertebral‐inspired architecture for structural strength, (2) a glass fiber‐nylon composite (30 wt% GF) ribcage‐mimetic shell engineered for impact resistance and controlled hygroscopic expansion, and (3) an intermediate PU interlayer (Shore 90A‐92A) providing viscoelastic damping and load redistribution capabilities. So, we have developed three distinct mechanical architectures: a bare vertebral‐inspired skeleton (F1), its PU‐infiltrated counterpart (F2), and an axial skeleton assembly with PU matrix coupling (F3), as systematically characterized in Figure 4b.

To evaluate the dynamic hydrostatic adaptability of the mechanical skeleton, we have measured the equivalent compressive moduli of F1‐F3 prototypes under pressure loading. Prior studies have demonstrated that elastomer‐rigid frame composites withstand material‐driven localized failure under 3 MPa hydrostatic pressure (300 m water depth) [25]. Given that the structural boundary condition differs from full‐ocean‐depth environment simulation device constraints [26], we have performed quasi‐static compression tests on three specimens, focusing on structural elastic deformation (Figure 4c). Following validation of the finite element analysis (FEA), we simulate hydrostatic loading from 1 to 6 MPa, corresponding to specimen F3's compression range of 0.453–2.379 mm. Based on normal distribution principles, the median‐proximal displacement of 1.66 mm serves as the representative compression magnitude (Figure 4d,e). Localized deformation effects under hydrostatic loading are systematically excluded to ensure experimental‐simulation consistency (Figure S4), which enables rigorous quantification of equivalent compressive modulus evolution within the linear elastic domain.

Experimental and numerical analyses demonstrate that the carbon fiber‐reinforced composite F1 achieves an equivalent compressive modulus of 94.28 MPa. Experimental measurements and finite element simulations agree closely, with differences of less than 2%. Under out‐of‐plane (*z*‐axis) compressive loading, carbon fiber tubes exhibit simultaneous through‐thickness compression and in‐plane (*xy*‐plane) expansion (approximately 4.8 mm). This deformation behavior is attributed to the unconstrained lateral boundaries at both ends of the tubular structure. This mechanical behavior mirrors structural scoliosis caused by muscular atrophy or hypotonia, where curvature deformations below 12% demonstrate self‐correcting potential. Implementing peripheral elastic PU reinforcement around the carbon fiber tubes effectively mimics the compensatory mechanism achieved through muscular strengthening in biological systems. In addition, the cross‐shaped plate integrated atop the central solid carbon tube enhances the load‐bearing surface area for planar compressive loading (94.1% increase). However, it may exhibit structural vulnerability to buckling instability under asymmetric loading conditions. These limitations are effectively addressed through strategic PU reinforcement implemented at the base interface. To optimize mechanical properties, the F2 composite architecture is developed by encapsulating the carbon fiber endoskeleton with elastic PU. This structure exhibits an equivalent compressive modulus of 96.68 MPa, achieving a 2.5% increase in mechanical robustness relative to the unenhanced endoskeleton system. While the mechanical properties show a modest 2.5% enhancement, the in‐plane expansion deformation decreases substantially (35%), resulting in improved stability. Experimental and simulation results demonstrate a 1.2% deviation, with the observed discrepancy remaining within experimental tolerance. However, as a nearly incompressible material, elastic PU undergoes pronounced in‐plane expansion when subjected to out‐of‐plane loading, particularly at free structural edges (approximately 3.6 mm). This strain‐induced geometric distortion substantially compromises structural robustness. Although the flexible‐shell structural array exhibits enhanced pressure adaptability, it struggles to maintain boundary integrity in complex underwater environments. Under deep‐water conditions, hydrostatic pressure effects may induce interfacial water permeation through structural gaps. To address these challenges, we develop a PU‐encapsulated external framework employing a biomimetic design strategy. Inspired by the human rib cage's multifunctional morphology, this configuration emulates the rib's organ protection, structural reinforcement, and muscle anchoring interfaces. This structural design imposes displacement constraints on the PU that counteract deformation vectors, thereby enhancing the material's out‐of‐plane load‐bearing capacity while improving the mechanical robustness of the composite system. The hydroxyl‐rich surface of GFs (─OH groups) enables hygroscopic absorption through hydrogen bonding with environmental moisture. This moisture‐induced hygroscopic expansion generates compressive stresses that promote self‐locking within the array architecture, thereby motivating the selection of nylon‐GF composites for structural fabrication. The equivalent compressive modulus of the overall configuration F3 is 100.08 MPa, and the error between experiment and simulation is 2.2%, within the error tolerance. In addition, structural optimization enhances the mechanical robustness of F3 by 3.4% compared to F2, accompanied by a 64.5% reduction in radial displacement under loading (Figure 4b). This refinement simultaneously achieves homogeneous circumferential strain distribution (Figure 4c) through decoupled deformation mechanisms: carbon fiber tube arrangement no longer governs global deformation patterns, while PU exhibits eliminated local stress concentrations.

In contrast, numerical simulations of F3 under hydrostatic pressure reveal distinct deformation characteristics compared to quasi‐static conditions (Figure 4d). The displacement contour plot exhibits strain localization patterns arising from mechanical heterogeneity between components, manifested as disparate displacement gradients across the compression interface. First, statistical median analysis demonstrates that the carbon fiber‐reinforced skeleton (F1) maintains elastic behavior under 1.66 mm compressive displacement, with experimental validation confirming the absence of plastic deformation or buckling failure modes. Discrete analysis reveals that under 1–6 MPa hydrostatic loading, F3's maximum displacements (0.497–2.559 mm) originate predominantly from the compliant PU matrix. The carbon fiber reinforcement composite exhibits minimal displacement (<0.15 mm, approximately 5% of total deformation), demonstrating nearly 100% structural integrity retention at 6 MPa through strain energy partitioning. Second, hydrostatic loading induces persistent in‐plane strain localization within the PU matrix, exhibiting anisotropic contraction patterns despite uniform pressure distribution. Stress predominantly localizes in regions with wider carbon fiber tube spacing (≥23.64 mm), where diminished PU support efficacy leads to localized compressive strain. Due to the material's almost incompressible properties, this out‐of‐plane compression phenomenon induces significant in‐plane lateral expansion through Poisson's effect. Quantitatively, the expansion displacement exhibits linear pressure dependence, increasing from 0.3 to 1.2 mm as hydrostatic pressure escalates from 1 to 6 MPa. Finally, hydrostatic pressure loading induces pronounced interfacial effects that dominate out‐of‐plane deformation patterns. Interface compression between rigid (excluding the nylon‐GF composite framework) and compliant materials remains negligible. However, out‐of‐plane displacement demonstrates progressive amplification along PU matrix propagation paths. Out‐of‐plane compression couples with in‐plane expansion at PU/nylon‐GF interfaces, particularly in carbon fiber‐depleted zones. This pressure‐dependent coupling elevates interfacial delamination risks as the hydrostatic loading increases. It is worth noting that the hydrostatic pressure‐induced localized strain is confined to superficial structural layers (approximately 0–2 mm depth) due to boundary constraints, exhibiting thickness‐dependent attenuation. Meanwhile, the hydrostatic pressure at 600 m depth induces ≤5% interfacial misalignment. While this marginally reduces sound attenuation capability (average Δ = 0.1 per 1 MPa), the PU matrix demonstrates nearly 100% shape recovery upon pressure relief, with pressure‐adaptive hysteresis enhancing structural reusability.

In summary, the composite structure (fiber‐reinforced skeleton and PU matrix) demonstrates exceptional mechanical robustness (Equivalent compressive modulus: 100.08 MPa) through bioinspired architectural design. The muscle‐mimetic elastic and contraction behaviors enable nearly 100% hydrostatic pressure adaptation capability, while the skeletal reinforcement generally confines local strain to <3%. This synergistic configuration produces nonlinear performance gains exceeding linear superposition predictions.

### 2.5 All‐in‐One Underwater Composite Structure Transforming Into an Applied Overlay Skin

Through rational material selection and structural reinforcement, we design gradient‐distributed steel masses within carbon fiber‐reinforced PU tubes. This configuration leverages local resonance mechanisms to establish spring‐mass systems that enable low‐frequency sound attenuation in parallel structures. In addition, building on impedance matching principles, following hydraulic principles observed in spider legs shown in Figure 2, we position a bioinspired heteromorphic structure beneath the hollowed‐out region of the central carbon fiber plate. This configuration exploits the structural deformation mechanism under hydrostatic pressure to amplify multidirectional mechanothermal conversion, effectively dissipating acoustic energy through internal thermal losses. Meanwhile, a microscale cavity integrated at the structural base exploits the gas‐solid impedance mismatch to induce wave‐mode conversion and effectively dissipate acoustic energy. The all‐in‐one requirements of mechanical robustness and acoustic conditioning are substantiated through two key analytical dimensions. Initially, comparative sound attenuation trends under hydrostatic pressure are used to achieve a balanced optimization of acoustic and mechanical performance. Furthermore, systematic variations in impedance modulation and wave velocity reconstruction across material combinations (Figure S3) reveal critical material‐structure‐property relationships. These results are related to the fundamental mechanical trends observed in Figure 3, where both elastic modulus and Poisson's ratio exhibit material‐specific progression patterns that directly influence sound wave propagation characteristics. Material selection is guided by on‐demand matching and performance trade‐off analyses. The 5‐mm‐thick surface layer and matrix made of PU (Shore hardness 90A) achieve optimal mechanical‐acoustic balance in the integrated underwater composite structure.

**FIGURE 5:**
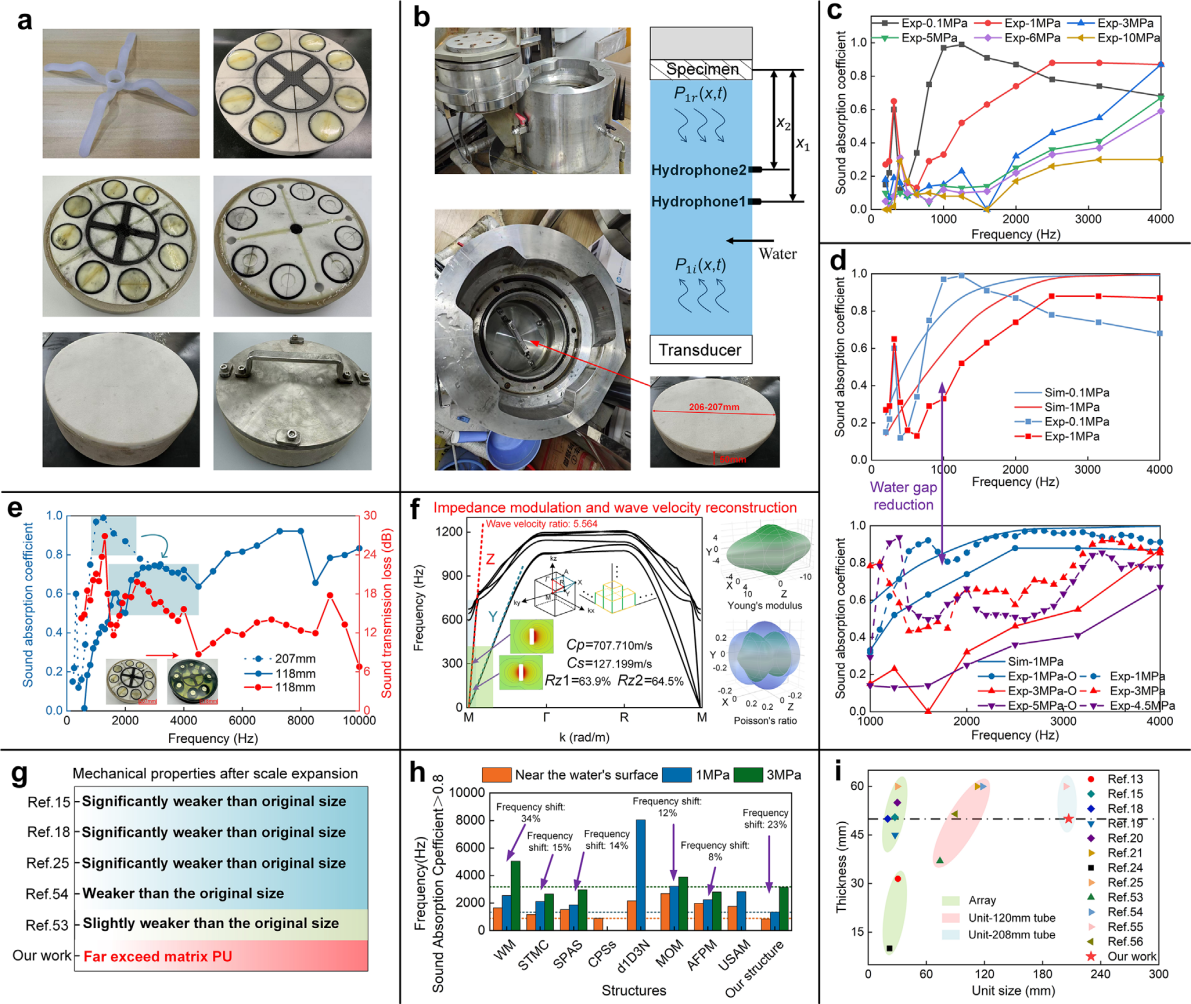
Pressure‐resistant sound absorption testing and all‐in‐one assessment of underwater composite structures. (a) Spider‐like heteromorphic structure and integrated configuration. (b) Underwater acoustic impedance tube and configuration schematic. (c) Hydrostatic pressure‐dependent sound absorption: experimental characterization (0.1–10 MPa). (d) Hydrostatic pressure‐dependent sound absorption: experimental vs. simulated comparison (0.1–1 MPa). Guided by analysis of simulation‐experiment error and sound absorption attenuation under hydrostatic pressure, the manufacturing process is optimized to enhance structural continuity and reduce water gap effects, thereby improving pressure‐resistant sound absorption performance. (e) A multi‐scale characterization of sound attenuation properties. It involves frequency shift phenomena arising from scale changes and sound insulation effects that complement sound absorption. (f) Irreducible Brillouin zone, dispersion curves, and tensor analysis. Among them, the usual tour of the wave vector *k* = (*kx*, *ky*, *kz*) through the first Brillouin zone. The bounds of the Brillouin zone are depicted as a cube. The paths are highlighted in red. Meanwhile, the pressure and shear wave velocities are characterized based on the direction of vibration. (g) Mechanical properties after scale expansion. (h) Comparison of the initial frequency at which the sound absorption coefficient exceeds 0.8 under different hydrostatic pressures. Reference data: WM [53]. STMC [54]. SPAS [25]. CPSs [21]. d1D3N [24]. MOM [19]. AFPM [55]. USAM [15]. (i) Unit size and thickness comparison [56].

While vacuum‐assisted rubber injection has proven effective for manufacturing monolithic PU specimens (<20 cm^3^), interfacial uniformity deteriorates in multilayer composites requiring sequential infusion, particularly in structures exceeding 30 cm^3^ per layer. Therefore, the structural matrix is fabricated via an additive manufacturing process following PU parametric optimization, ensuring material homogeneity and dimensional fidelity in each component. The fabricated sample is shown in Figure 5a. For acoustic characterization, the sample is mounted in an impedance tube (Figure 5b) with an inner diameter of 208 mm. Acoustic properties are evaluated across 0.2–4 kHz.

As shown in Figure 5c, the sound attenuation performance is assessed under hydrostatic pressures ranging from 0.1 to 10 MPa, with the corresponding data systematically presented. The absorption spectrum demonstrates a pressure‐dependent shift, evidenced by the structure's effective bandwidth progressively shifting to higher frequencies with increasing hydrostatic pressure. Within the operational range (0.2–4 kHz), the sound attenuation peak (>0.8) persists only below 3 MPa, above which attenuation efficiency declines relatively significantly. At 0.1 MPa hydrostatic pressure (simulating near‐surface conditions), the structure achieves near‐perfect sound absorption (*α* = 0.99) at 1.25 kHz, with 0.8–2.5 kHz bandwidth sustaining coefficients above 0.8. This dual capability of peak perfection and broadband efficacy establishes benchmark performance for low‐frequency underwater sound attenuation. Meanwhile, at 1 MPa hydrostatic pressure (equivalent to 100‐meter depth in aquatic environments), the structure exhibits a sound absorption peak (α near 0.9) at 2.5 kHz while maintaining broadband efficacy (*α* > 0.8) across 2.2–4 kHz. Analysis reveals the structure maintains broadband acoustic performance under hydrostatic loading, preserving a 1.7 kHz bandwidth with *α* > 0.8, while exhibiting an 11% reduction in peak absorption coefficient (0.99–0.88) compared to ambient pressure conditions. This coupled response demonstrates pressure‐independent broadband stability coexisting with peak sensitivity to hydrostatic pressure. When the hydrostatic pressure reaches 3 MPa, the effective absorption band shifts to 4 kHz. After exceeding 3 MPa, the acoustic performance exhibits progressive degradation with increasing pressure. The average sound absorption coefficient is quantified by a nearly 4.5% reduction in hydrostatic pressure per 1 MPa increment, demonstrating pressure‐dependent low‐frequency sound absorption losses in the structural matrix. Post‐experiment analysis reveals structural recovery (≥90%) in the sample after returning it to atmospheric pressure following hydrostatic pressure loading. Then, achieve nearly 100% deformation recovery within 24 h. This reversible behavior demonstrates exceptional pressure‐adaptive resilience with recoverable acoustic performance, indicating potential for reusable underwater acoustic applications requiring cyclic pressure endurance.

We numerically analyze the sound absorption performance of the structure under hydrostatic pressures ranging from 0.1 to 1 MPa, using the boundary conditions shown in Figure 2c. The simulation results demonstrate good agreement with experimental measurements, as quantitatively compared in Figure 5d. Meanwhile, we employ the TMM with acoustic‐electrical analogy to eliminate nonlinear geometric relationships between structural components, reducing discrepancies with theoretical results by 48.2%. A systematic theoretical framework is developed to calculate impedance across series‐parallel units and derive sound absorption coefficients, with full methodological details provided in Figure S1. The results show that experimental and simulated absorption curves exhibit strong trend consistency across hydrostatic pressures (0.1–1 MPa), with cutoff frequency errors in the low‐frequency absorption coefficient rise band remaining below 0.5%. The absorption coefficient exhibits a mean relative error of ∼10% at 0.1 MPa hydrostatic pressure, whereas increasing the pressure to 1 MPa reduces this error to 6.92%. The values are within the tolerance error, verifying the accuracy of the simulation method. Three primary error sources are identified: (1) The hygroscopic expansion of additively manufactured PU matrices induces anisotropic swelling under structural confinement, causing parameter variations at multi‐material interfaces that lead to impedance mismatch differences and subsequent reduction in sound absorption coefficient. (2) Boundary water gaps form due to sample‐tube diameter mismatch, introducing measurement errors. Under increasing hydrostatic pressure, fabrication‐induced lateral boundary discontinuities propagate water gaps, thereby modifying the structural loading and constraint conditions. (3) Interfacial defects arise between components during structural fabrication. Sound waves impinging on structural surfaces induce partial longitudinal‐to‐transverse wave conversion, thereby exciting structural vibrations. The 3614.2:1 water‐air characteristic impedance mismatch induces amplitude differentials and phase distortions through multi‐degree‐of‐freedom coupling with nonlinear hysteresis, generating interfering pseudo‐acoustic sources that degrade hydrophone signal fidelity. Based on the identified error sources, the fabrication process is optimized. Sealing layers of PU or epoxy resin are applied at rough internal interfaces and at the lateral junctions between the external PU top plate, steel backing, and the main structure. This treatment reduces internal defects and improves lateral interface continuity. Consequently, unnecessary sound scattering is minimized, and the impact of water gaps is mitigated. Supplementary experimental results reveal that the sound absorption curves retain their original trend while exhibiting a substantially increased coefficient. Under 1 MPa hydrostatic pressure, the experimental and simulated curves closely coincide, confirming the validity of the error analysis. Additionally, the structure achieves an average sound absorption coefficient of 0.872 under 1 MPa hydrostatic pressure and maintains it above 0.7 under 3 MPa within the tested band, further demonstrating its advantage in low‐frequency broadband sound attenuation.

The sound attenuation performance of underwater structures can be evaluated by determining their sound absorption coefficient and transmission loss in aquatic environments. These parameters are derived from solutions to the sound reflection and transmission coefficients, which collectively characterize the structure's capability to attenuate propagating sound waves through energy dissipation and storage effects. Among them, the sound reflection coefficient is determined as follows:

(2) $$r_{p}=\frac{Z_{s}-\rho_{w}c_{w}}{Z_{s}+\rho_{w}c_{w}}$$

where the water density ρ_*w*_ = 1 g/cm^3^, the sound velocity c_*w*_ = 1500m/s, and the water impedance Z_w_ = ρ_*w*_ c_*w*_. Meanwhile, the overall surface impedance of the sample is *Z_s_*. The structural sound absorption coefficient (α) is derived from these parameters.

(3) $$\alpha =1-{\left|r_{p}\right|}^{2}$$

In contrast to sound absorption, which primarily involves energy dissipation through impedance effects, sound transmission loss predominantly arises from sound wave incident /transmission and mechanical energy storage during structural vibration. The transmission coefficient is calculated from the incident and transmitted sound intensities, defined as:

(4) $$t_{p}=\frac{\frac{{\left|P_{\mathrm{ta}}\right|}^{2}}{Z_{2}}}{\frac{{\left|P_{\mathrm{ia}}\right|}^{2}}{Z_{1}}}$$

where *P_ia_* and *P_ta_* are the sound pressure amplitudes in the incident and transmitted sound fields, respectively. Z_1_ and Z_2_ are the characteristic impedances of the medium above and below the structure. The structural sound transmission loss (STL) is derived from these parameters.

(5) $$\mathrm{STL}=10\mathrm{lg}\frac{1}{t_{p}}$$

The limited diameter of the impedance tube restricts the measurement of broadband sound insulation for large‑scale structures. It also limits the evaluation of sound absorption performance at frequencies above 4 kHz. To investigate the broadband sound attenuation capability of the structure in greater depth, a scaled‑down concept is adopted. The structural dimension is reduced to 118 mm to accommodate a 120 mm diameter impedance tube. This method enables exploration of the relationship between the scaling factor and the resonance/anti‑resonance frequencies. This mapping relationship can also be used to reverse derive the broadband sound attenuation trends of large‑scale configurations under ideal conditions. This capability further refines the research framework. The sample preparation and testing process is illustrated in Figure S6, and the corresponding results are presented in Figure 5e. A comparison of the peak absorption frequencies between the large‐ and small‑scale structures reveals a shift toward higher frequencies in the smaller configuration. This shift factor is essentially consistent with the applied scaling factor. Meanwhile, the structure exhibits a broadband sound absorption capability, with a coefficient exceeding 0.6 over a bandwidth of 7.9 kHz. Furthermore, it's exceeding 0.7 over 6.2 kHz. Although structural impedance mismatch with water is intensified by thickness shrinkage (50–30 mm), variations in matrix forming processes, and manual fabrication errors. Consequently, the overall sound absorption coefficients decrease. Nevertheless, higher‑frequency sound absorption surpasses its low‑frequency counterpart in the small‑scale configuration. This observed trend enables the inference of the broadband sound attenuation behavior of the 207 mm large‑scale structure, thereby validating its acoustic performance. On the other hand, the scaled‑down structure exhibits a low‑frequency sound insulation advantage. At 1.3 kHz, it attains a transmission loss of 26.87 dB. This low‑frequency effect is triggered by structural anti‑resonance arising from a local resonance mechanism. At this frequency, the average out‑of‑plane displacement of the structure approaches zero, while the equivalent stiffness reaches its maximum. As a result, sound waves cannot effectively excite contraction or expansion of the structure and are predominantly reflected. Furthermore, the anti‐resonance frequency of the 207 mm structure is estimated to be nearly 800 Hz, based on reverse derivation from the scaling factor. Its increased thickness enhances sound insulation by contributing to greater mass. The low‐frequency sound insulation performance of large‐scale structures can also be further enhanced. The structural discretization results from numerical calculations [48] are presented in Figure S6d. The above numerical results demonstrate good agreement with the experimental data. Errors remain within the permissible range, thereby validating the accuracy of the simulation method.

The circular geometry is topologically converted to a square configuration while preserving the effective vibrational area and unit‐cell periodicity. Phonon band structure along the usual tour of the wave vector $\vec{k}$ = (kx, ky, kz) through the high‐symmetry points of the first Brillouin zone of the simple‐cubic lattice is shown in Figure 5f [49]. Among them, the red‐highlighted trajectories are the primary paths of interest, which enable a more accurate representation of the vibrational response and wave transfer patterns compared to the secondary paths (blue trajectories). Modal analysis identifies the fundamental transverse and longitudinal modes, corresponding to the lowest non‐zero frequencies in the dispersion curves [50, 51, 52]. The group velocity, calculated as the linear slope within the low‐frequency passband (f < 1 kHz), determines the effective wave velocity. Meanwhile, the transverse (Cs) and longitudinal (Cp) wave velocities are used to calculate the anisotropy ratio and the critical scale ratio. These ratios characterize the structure's wave‐scattering performance (Scatter nephogram as shown in Figure 5f). We also analyze the impedance modulation effect. This involves evaluating the impedance match between the structure's surface and water, as well as the structure's equivalent impedance (modeled as a skin covering) and that of water. This optimized parameter combination enables the manipulation of low‐frequency sound waves. By guiding their energy into omnidirectional scattering, it substantially reduces the intensity of backscattered echoes. However, acoustic stealth inherently conflicts with mechanical load‐bearing due to all‐in‐one requirements. Structural reconfiguration achieves optimal performance by balancing these competing objectives. As shown in Figure S3, the logic‐gate‐based reconfiguration framework processes the constituent material parameters. It evaluates the wave velocity anisotropy levels and impedance modulation capabilities of different structural variants. The experimental structures (Figure 5a–d) have achieved optimal performance: low‐frequency diffuse reflection (wave velocity ratio: 5.564, Shear (S) wave wavelength ≥ 2π characteristic size, and exhibits a transition from Rayleigh scattering to Mie scattering at low frequencies), low‐frequency broadband sound attenuation (0.8–2.5 kHz, *α* > 0.8), and high mechanical robustness (matrix‐skeleton equivalent compressive modulus exceeds 100 MPa, with nearly 100% deformation recovery).

Finally, the multi‐objective performance of our structure is benchmarked against prior works, highlighting its advantages in three key aspects: load‐bearing capacity based on scale, sound attenuation under hydrostatic pressure, and larger unit‐cell dimensions (Figure 5g–i). From the perspective of unit‐cell scale and load‐bearing performance, many existing designs integrate small‐scale periodic arrays with rigid supporting frameworks. This strategy provides adequate load‐bearing capacity when unit dimensions are small. Under hydraulic pressure, a smaller frame size reduces the maximum out‐of‐plane displacement of the internal elastic material, thereby mitigating the shift of the effective sound‐attenuation band toward higher frequencies. Furthermore, a reduced frame dimension is more effective in minimizing overall deformation. However, small‐scale structural arrays, while theoretically feasible, face practical limitations in cost, design flexibility, and scalability in real‐world engineering applications. In contrast, designs incorporating internal skeletons are less constrained by size and maintain excellent mechanical and acoustic performance even when scaled up.

### 2.6 Energy Weak Entanglement and Impedance Strong Mismatch Mechanisms

Analysis of the structure's low‐frequency sound attenuation reveals two favorable factors that break the routine: weak kinetic‐potential energy entanglement and strong impedance mismatch. This attenuation mechanism is explained by analyzing acoustic resistance, acoustic reactance, energy storage, energy dissipation, and parametric dependencies.

**FIGURE 6:**
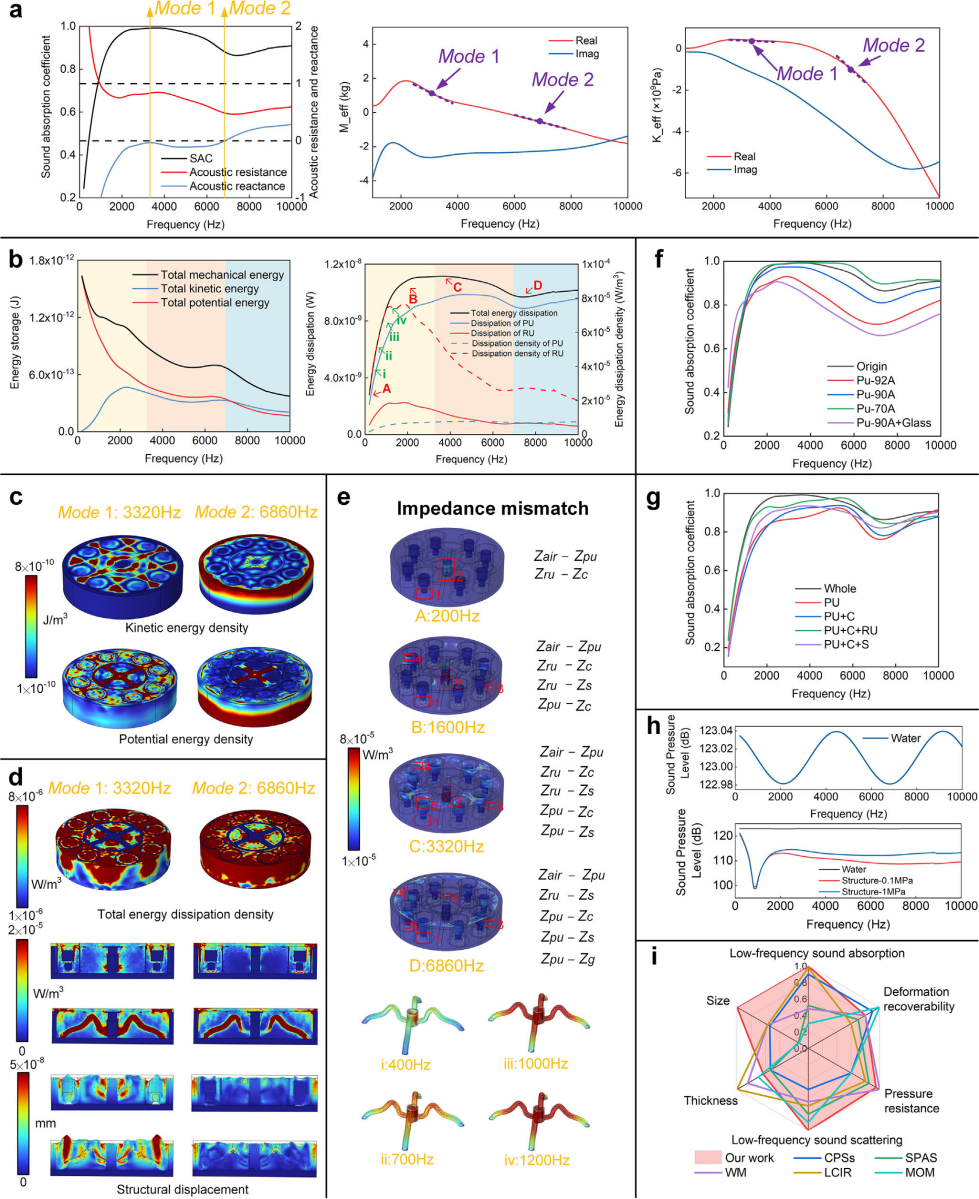
Energy‐ and impedance‐based characterization of sound attenuation mechanisms. (a) Sound absorption coefficient at 0.1 MPa, with corresponding acoustic impedance curves. The acoustic resistance and reactance curves can be determined from a further analysis of the equivalent mass and stiffness of the structure. Their combined analysis reveals the sound absorption mechanism. (b) Analysis of energy conversion and per‐material component energy dissipation under sound wave propagation. Among them, kinetic‐potential energy entanglement consists of an acoustic‐to‐mechanical energy conversion process; low‐frequency dissipation from acoustic impedance mismatch in material components. (c) Kinetic and potential energy distribution at transition frequencies across three stages. (d) Energy dissipation and displacement distribution of constituent components at transition frequencies across three stages. (e) Frequency‐dependent acoustic impedance mismatch law and hydraulic transmission‐inspired spider‐like heteromorphic structure energy dissipation process. (f) Material parameters gradient in PU variants governs sound attenuation performance. (g) Composition‐dependent sound absorption in structural materials. (h) Hydrostatic pressure‐dependent sound attenuation with metamaterial skin. i. Multi‐performance comparison of underwater composite structure. Reference data: CPSs [21]. SPAS [25]. WM [53]. LCIR [13]. MOM [19].

Within the range of 10 kHz, structural sound absorption progresses through three stages (enhancement stage, attenuation stage, and steady stage), driven by the coupled effects of three mechanisms: (1) weak kinetic‐potential energy coupling, (2) strong impedance mismatch, and (3) mechanical energy substantially converted to thermal energy dissipation. Figure 6a,b shows the sound absorption coefficient rising steeply to the peak below 3.32 kHz (α ≈ 0.99), achieving near‐total absorption. Total energy decreases by 46.2% in this frequency (3.32 kHz) through thermal dissipation: 24.9% converts to kinetic energy transmission, and 28.9% converts to potential energy storage. From the material point of view, broadband absorption primarily relies on PU material's dissipative capacity. However, rubber contributes more prominently during the first stage, exhibiting 13.2× greater peak energy dissipation density than PU. The above phenomenon indicates the low‐frequency sound attenuation advantage of the internal rubber material. Multi‐material coupling enhances sound absorption through two mechanisms: macroscopic interfacial frictional heating from efficient energy conversion, and microscopic molecular thermal vibrations. This synergy yields near‐ideal acoustic properties (resistance close to 1, reactance close to 0).

Notably, the first stage exhibits an extremum in kinetic‐potential conversion rate at approximately 3.32 kHz. However, the value discrepancy between kinetic (4.07 × 10^−13^J) and potential energies (4.73 × 10^−13^J) persists, unrealizing perfect resonance (acoustic reactance < 0). At this frequency, neither the structural equivalent mass nor the stiffness has passed through zero, exhibiting relatively low values. The inertial response decreases, while stiffness gradually increases. As a result, the elastic energy storage mechanism becomes the primary contributing factor. Within this frequency band, the dissipation mechanisms associated with inertia and stiffness (the imaginary part) reach a dynamic equilibrium, which stabilizes the normalized impedance near unity and thereby dissipates a substantial portion of the system energy. Consequently, the resonance remains obscured by dissipative processes, including material damping, radiation loss, and molecular thermal vibration. Concurrently, the resonance peaks are suppressed and broadened. This effectively extends the operational bandwidth (1.78–4 kHz) while maintaining high peak values (*α* > 0.95). From the energy perspective, this behavior is attributed to low‐frequency weak entanglement, arising from the low impedance and high damping characteristics of the spider‐like heteromorphic structure (Figure 6b).

Specifically, energy dissipation density distributions at positions i–iv (Figure 6b,e) reveal that dissipation extends from the center to the periphery in the spider‐like heteromorphic structure. This design, inspired by viscous hemolymph dissipation during Salticidae jumping, strategically positions damping elements beneath the cross‐shaped plate, fixed by a carbon column. The configuration provides structural support and enhances material interactions, thus facilitating effective kinetic and potential energy transfer. Meanwhile, Figure 6e reveals an inverse relationship between impedance mismatch magnitude and dissipation frequency: larger impedance gaps correspond to lower‐frequency energy dissipation (positions A–D). The rubber's impedance (one order of magnitude below the PU matrix) lowers the mechanical‐thermal conversion frequency at material interfaces to ∼2 kHz. This enhances potential energy storage (via vibration) and thermal dissipation throughout the system. The above effect causes the frequency of kinetic‐potential entanglement to shift to lower frequencies, contributing to the low‐frequency sound attenuation performance of the structure.

The energy entanglement phenomenon initiates at 2 kHz and persists through the second stage. We thus focus on Mode 1 frequency (characterizing energy weak entanglement) to quantify its contribution to the structure's sound attenuation performance. Figure 6c–e shows kinetic energy maxima concentrated in the PU matrix adjacent to the mechanical skeleton at 3.32 kHz. Simultaneously, potential energy peaks localize within matrix regions between carbon fiber tubes. Multiple structural sub‐modes are excited simultaneously. The displacement exhibits a state dominated by local resonance with anti‐phase and transverse‐longitudinal coupling. Different elastic responses cancel each other out due to phase opposition. Meanwhile, the structure nearly loses its macroscopic inertia. At this frequency, acoustic energy is difficult to store, ultimately dissipating as thermal energy. Furthermore, the energy dissipation and displacement distribution reveal significant dissipation at material junctions in the different components. As point C in Figure 6e confirms, this originates from wave‐mode conversion due to impedance mismatch. Waves propagate through structures and form specific vibrational energy distributions, owing to uneven internal impedance and boundary reflections. Significant relative motion tends to occur in regions of high damping (PU, rubber) or at interfacial connections (PU‐carbon fiber, PU‐steel, PU‐air, rubber‐carbon fiber, and rubber‐steel). This motion excites highly damped elastic materials, inducing intense shear deformation that activates their inherent shear hysteresis. Consequently, acoustic energy is efficiently dissipated as thermal energy via mechanical work. Due to pronounced interfacial friction and viscoelastic dissipation, the accumulation of kinetic energy in the system is significantly suppressed, allowing potential energy to dominate. It continuously interrupts the energy storage process within each oscillation cycle. As a result, the acoustic reactance approaches zero, yet does not strictly reach zero. In summary, the structure leverages its damping and impedance characteristics to induce kinetic‐potential energy weak entanglement. This facilitates effective low‐frequency sound attenuation through three synergistic dissipation pathways: (1) local resonance friction, (2) wave‐mode conversion, and (3) molecular thermal vibration.

At the Mode 2 resonance (6.86 kHz), the weak kinetic‐potential energy entanglement transitions to full coupling. This enables weak material‐damping correlation resonant action (acoustic reactance = 0). Refer to Figure 6a,b, the inertia‐related dissipation gradually declines with rising frequency, while the stiffness‐related dissipation increases continuously. This combined effect causes the overall acoustic resistance to drop and diverge from the matched condition. Simultaneously, the resonant frequency exhibits an attenuation of thermal dissipation (Figure 6b), which is directly linked to the acoustic resistance offset. Combined with Figure 6c,d, resonance primarily manifests as synergistic coupling between the matrix and nylon‐GF axial skeleton. Displacement is concentrated predominantly on the upper side of the spider‐like heteromorphic structure and within the frame region, where the peak energy dissipation also concentrates at these locations. Although the spider‐like heterostructure and the frame displace spatially in the same direction along the sound‑wave incidence, the substantial phase lag arising from the soft connections produces a pronounced dynamic anti‑phase response. Weakened damping (primarily reduced rubber's viscoelastic loss) diminishes mechanical‐to‐thermal energy conversion efficiency, thereby degrading sound absorption. Furthermore, at the third stage, positive acoustic reactance confirms mass dominance, yielding kinetic‐energy‐governed energy transmission. Within this frequency range, structural acoustic resistance rebounds post‐resonance. Friction at the PU‐based viscoelastic material/rigid frame interface subsequently dissipates the stored mechanical energy as thermal energy. This vibration‐dominated mechanism (kinetic energy‐vibration velocity), combined with multi‐material impedance mismatch, produces a weakly damped thermal dissipation (the energy dissipation effect of rubber decreases at high frequencies). Together, they establish the primary energy dissipation pathway for the third stage of sound attenuation.

Furthermore, parametric analysis of matrix material hardness shows that varying hardness can effectively modulate the structural equivalent impedance. Relative to the selected Shore 90A PU matrix, the 70A PU offers improved sound attenuation performance. However, this reduction in hardness significantly limits the material's suitability for deep‑water engineering applications. Meanwhile, increasing the matrix stiffness shifts the effective sound attenuation band toward higher frequencies. However, this trend is readily influenced by the material's Poisson's ratio. As shown in Figures 3c,d and 6f, adding hollow glass microspheres to 90A PU significantly reduces the Poisson's ratio while preserving the matrix hardness. This reduction shifts the effective sound attenuation band toward lower frequencies. Concurrently, sound absorption is diminished due to impedance mismatch with water.

We next demonstrate the performance advantages of underwater pressure‐resistant composite structures through three key aspects: structural configuration, sound pressure level attenuation, and comparative studies involving multiple teams. The composite structural system integrates two material classes: viscoelastic components (PU, rubber) and rigid components (carbon fiber, steel, nylon‐GF composite). While maintaining fixed structural dimensions, all components undergo adjustment within the PU matrix and nylon‐GF framework. The base structure features a PU matrix with an integrated nylon‐GF axial skeleton. Subsequent optimization constructs position‐specific material‐structure numerical models. Figure 6g shows consistent sound absorption trends between the base and final configurations. The difference lies in the magnitude values, with a variation of approximately 13%. Among them, the carbon fiber skeleton confines vibrational motion in the PU matrix. This confinement creates an acoustic impedance mismatch at the interface, dissipating acoustic energy through friction‐induced thermal losses. Consequently, the composite exhibits enhanced sound absorption. Meanwhile, the spider‐like heteromorphic structure, incorporating strategically placed damping material, exhibits superior sound absorption compared to the locally resonant design with rigid inclusions. Its absorption bandwidth exceeds that of the resonant design by >15%, while the peak absorption value surpasses it by >5%. This enhanced low‐frequency attenuation arises primarily from the greater acoustic impedance contrast between rubber and carbon fiber. Simultaneously, the damping material broadens the effective bandwidth. The synergistic action of dual mechanisms yields a composite structure with superior broadband attenuation performance.

Furthermore, we further quantify the underwater system's sound attenuation performance after applying an acoustic skin to its exterior. These results, characterized by sound pressure level reduction, are shown in Figure 6h. Given environmental complexities, this research focuses solely on overarching trends. The numerical results align with the sound attenuation trend in Figure 5e, demonstrating the pressure‐resistant composite structure's effective acoustic performance. To simulate practical environments accurately, we preserve the coupling between sound absorption and insulation properties. The acoustic assessment thus accounts for material damping effects (including coupled sound absorption). Ultimately, without the composite structure, sound pressure levels present simple harmonic dynamics near 123 dB. When applied, the structure induces gradual yet strong attenuation below 1 kHz (coupled sound scattering‐isolation effect) and sustained attenuation above 1.8 kHz (coupled sound absorption‐insulation effect).

Collectively, multi‐team analyses confirm the proposed structure's integrated advantages: optimized size and thickness, enhanced low‐frequency sound absorption and scattering, as well as exceptional mechanical robustness (Figure 6i). Structurally, this design replaces traditional small‐scale honeycomb units with large‐scale acoustic metacells reinforced by mechanical skeletons. This approach overcomes hydrostatic pressure limitations on underwater structures while enhancing tunability. By achieving synergistic multifunctional optimization, it establishes a scalable framework for acoustic skin design and application. Furthermore, this sub‐wavelength structure (just 50 mm thick) achieves broadband low‐frequency sound attenuation. This performance stems from two innovations: a damping‐material‐filled matrix and a heterostructured design that maximizes damping and impedance mismatch in conjunction with the surrounding skeleton. Simultaneously, the bioinspired muscle‐skeleton system enables hydrostatic adaptation. This facilitates multidimensional tunability in large‐scale units, significantly enhancing structural design flexibility.

## 3 Discussion

We present a multifunctional underwater acoustic skin integrating mechanical robustness, broadband sound attenuation, and low‐frequency diffuse reflection. We characterize the pressure‐driven deformation of PU‐based and hydrogel‐based materials using a dual‐stage selection platform. Unloaded, PU compressive modulus increases with hardness, while its Poisson's ratio decreases. Incorporating glass microspheres significantly reduces the Poisson's ratio of PU. Under progressive stress loading, PU exhibits a surge in compressive modulus at strains exceeding 1%, and its Poisson's ratio gradually increases toward a stable asymptotic value. In contrast, hydrogels show poor mechanical performance, characterized by a low compressive modulus and susceptibility to brittle fracture. Defects formed during this fracture process substantially reduce compressive strength. However, the advantageous sound attenuation of hydrogels, attributable to their very low S‐wave velocity and water‐like impedance, warrants consideration despite mechanical limitations. These findings establish a logical framework for analyzing material performance under pressure guidance. Inspired by the biomechanics of the human skeleton, we have designed a pressure‐adaptive mechanical skeleton that mimics muscle‐bone interactions. This design abandons traditional honeycomb structures and leverages the nearly incompressible deformation behavior of PUs. The skeleton exhibits pioneering mechanical stability, with an equivalent compressive modulus exceeding 100 MPa and pressure‐dependent local deformation generally below 3%. It also demonstrates outstanding deformation recoverability, approaching 100%. These properties enable the structural units to overcome the size limitations inherent in honeycomb‐reinforced structures and achieve significantly enhanced mechanical robustness. Further inspired by hydraulic transmission and viscous hemolymph dissipation in Salticidae, we have designed acoustic absorber components exhibiting strong impedance mismatch and high damping characteristics. Combined with structural anisotropy, this approach enables comprehensive broadband sound absorption and low‐frequency diffuse reflection, achieved through logic‐gate screening. Concurrently, we establish a quality visualization evaluation system. This system integrates an equivalent circuit model optimized by acoustic‐electrical analogy, allowing intuitive functional evaluation of the structure. Nevertheless, the current experimental characterization is limited to frequencies below 4 kHz. Consequently, high‐frequency performance requires numerical assessment. Similarly, the established quality evaluation system currently serves as a demonstrative case study, specifically applied to assess the sound absorption of the parallel unit containing spider‐like heterogeneous structures. Future research will address these limitations through investigations into multi‐scale unit integration and enhanced multifunctionality.

In general, this work provides an integrated acoustic‐mechanical multifunctional solution and establishes a corresponding quality evaluation system. By synergizing material‐structural properties and biomimetic strategies, proposing a novel pressure‐adaptive concept, we overcome structural unit scale limitations to enable synergistic enhancement of sound attenuation, diffuse reflection, and mechanical robustness. The former resolves the long‐standing trade‐off between pressure resistance and sound attenuation in traditional cladding layers, while the latter pioneers the characterization of pressure‐dependent material properties and structural enhancement synergies. Collectively, this advances a coupled design paradigm for low‐frequency broadband multifunctional stealth materials that better simulate real environments while enabling visual functional evaluation.

## 4 Methods

### 4.1 Sample Fabrication and Characterization

PU‐based composites and hydrogels are prepared based on a dual‐phase systematic investigation framework. Among them, PU‐based composites are prepared by first mixing equal proportions of soft PU‐A and soft PU‐B, corresponding to the desired Shore hardness. Air bubbles entrapped during mixing are removed via vacuum degassing. Subsequently, the degassed mixture is cast into molds and cured in a temperature‐controlled chamber for 72 h. Similarly, glass microsphere‐reinforced composites are fabricated. Here, a predetermined ratio of glass microspheres is incorporated during the initial mixing of soft PU‐A and B. The subsequent processing steps‐ degassing, pouring, and curing‐ are identical to those described for the PU‐based composites. Furthermore, the 14 wt% PAAm hydrogel is prepared as follows: First, 10 mL of 14 wt% acrylamide (AAm) monomer solution is placed in a beaker. Subsequently, 50 µL of 0.1 mol/L MBAA crosslinker, and 50 µL of 10 wt% ammonium persulfate (APS) thermal initiator are added. To accelerate curing, 50 µL of 10 wt% N, N, N', N'‐tetramethylethylenediamine (TEMED) is introduced as an accelerator. The mixture is thoroughly mixed, cast into molds, and cured at 60°C for 1 h in a temperature‐controlled chamber [15]. Material properties correspond to Table S2.

Following the specifications outlined in ISO 7743: 2007 for standardized mechanical testing protocols, we have fabricated cylindrical samples with nominal dimensions of 12.5 mm in height and 29.5 mm in diameter. The specimen geometry is maintained within ±0.1 mm tolerance to ensure dimensional consistency across all experimental replicates. To account for manufacturing tolerances, specimen dimensions are validated through triplicate measurements using a calibrated digital micrometer (0–12.7 mm range) and an analytical micrometer.

Furthermore, in terms of structures, the composite structure employs a four‐part modular design during additive manufacturing, also creating precision spaces for embedding carbon fiber‐reinforced tubular resonators and the spider‐like heteromorphic structure. Central truncation of the PU matrix inside the carbon tube maintains predefined voids for steel counterweights and cavities. The remaining components are fabricated through stereolithography (SLA) and selective laser sintering (SLS). Meanwhile, high‐precision shaping is achieved through computerized numerical control (CNC) milling, waterjet cutting, and related processes, with subsequent assembly achieving interfacial tolerances <1 mm, as detailed in Figure S5.

### 4.2 Simulation Methods and Setups

Acoustic part: A finite element model is constructed in COMSOL Multiphysics 6.0 using the Acoustic‐Solid Interaction module. For sound absorption evaluation, an incident sound field is applied on one side of the structure with a rigid boundary (total reflection) on the opposite side. Conversely, sound insulation assessment requires incident and transmitted sound fields on both sides to quantify structural attenuation. Specified displacement boundary conditions simulate the impedance tube constraints; meanwhile, a plane wave excitation uses a 1 Pa pressure amplitude. Furthermore, absorbing boundaries (Perfect matching layer: PML) applied to water domains emulate infinite fields without reflection.

Vibration part: Bloch‐wave analysis quantifies dispersion relations and wave velocities in periodic structures. This is implemented via a COMSOL Multiphysics numerical model using Bloch‐Floquet periodic boundary conditions within the Solid Mechanics module. Since the bounds of the first Brillouin zone are depicted as a cube, the paths are highlighted in Figure 5f. The first six vibrational modes are extracted, and the first three are focused on to characterize longitudinal and transverse wave velocities at the unit‐cell level. Subsequently, we characterize the anisotropy degree using the longitudinal‐to‐transverse wave velocity ratio. The structural equivalent impedance, calculated in combination with the equivalent mass density, quantifies the impedance modulation capability. This approach enables a comparison of the sound diffuse reflection performance among single units.

Mechanical part: We have analyzed the mechanical responses of the structure under quasi‐static compression and hydrostatic pressure using ABAQUS (version 2020; Dassault Systemes Simula Co., USA). To address convergence issues from large‐scale iterations, an explicit solution method is employed. For quasi‐static compression, the upper and lower platens are modeled as discrete rigid bodies. The lower platen is fully fixed, while the upper platen is constrained in all directions except vertically. Furthermore, hydrostatic pressure loading is simulated by uniformly applying pressure to the structure's upper surface. Mesh sensitivity analysis determines an optimal average element size of 0.2 mm. Meanwhile, the structure is discretized using eight‐node, 3D‐reduced‐integration solid elements (C3D8R).

### 4.3 Quasi‐Static Compression and Hydraulic Volumetric Change Rate Tests

In the testing of elastic material constitutive parameters, we have fabricated compression test molds according to ISO 7743: 2007 specifications and performed replicate mechanical testing using an electromechanical universal testing machine and hydraulic testing systems.

In terms of materials, quasi‐static compression tests are conducted on standardized specimens of PU‐based composites and hydrogels, following ISO 7743: 2007 specifications. A CMT5015 microcomputer‐controlled electro‐mechanical universal testing machine tests the higher‐modulus PU composites, while a TM2101‐T7‐01‐G single‐arm 2 kN universal material testing machine is used for the lower‐modulus hydrogels, based on their respective experimental load requirements. In addition, volumetric compression ratio measurements are performed using a hydraulic test system (built by Wuhan Wuce Test Technology Co., Ltd.) to determine the materials' Poisson's ratio under applied pressures ranging from 2.5 to 15 MPa.

Structurally, quasi‐static compression experiments are performed on three composite mechanical skeleton structures with varied configurations using an Instron 5982 universal testing machine equipped with a 100 kN load cell, following the ASTM C365 standard [30] (Figure 4c). The compression strain rate is maintained below 10^−3^ s^−1^ to ensure accuracy. Longitudinal displacement and the corresponding reaction force, measured by the testing machine, are used to calculate the average normal stress and nominal strain.

### 4.4 Scanning Electron Microscope Tests

To systematically investigate the root causes of material parameter discrepancies arising from variations in Shore hardness (70‐92A), glass microsphere filler concentration (9 vol%), and preparation processes, we have conducted comparative scanning electron microscopy (SEM) on PU‐based matrices with mechanically controlled microstructural features. Material samples are sectioned into slices (<1 cm) and dried. Conductive samples are used directly, while non‐conductive ones are gold‐coated to ensure conductivity. Subsequently, their microstructure is characterized by an SEM (JSM‐6480A) at an acceleration voltage of 0.5–30 kV.

### 4.5 Underwater Impedance Tube Tests

The hydrostatic pressure‐dependent sound attenuation performance of underwater composite structures is quantified using an acoustic impedance tube (ISO 10534 compliant). Samples are mounted in the impedance tube (208 mm inner diameter) to evaluate their sound absorption properties (Figure 5b). Measurements are performed over 0.2–4 kHz under hydrostatic pressures ranging from 0.1 to 10 MPa. Meanwhile, consistent with standardized acoustic testing protocols requiring simulated infinite impedance boundaries, a 10 mm‐thick steel backing plate (precision‐turned to flatness ≤0.02 mm) is mechanically bonded to the substrate base interface, effectively terminating structural vibration transmission paths. To evaluate broadband sound absorption and insulation, the scaled‑down structure is tested using a 120 mm diameter impedance tube. All measurements are performed within the 0.5–10 kHz frequency range. For sound insulation testing, the specimen is positioned at the middle of the impedance tube, with two hydrophones placed on each side to measure the sound transmission coefficient [57]. For sound absorption testing, the specimen is mounted at the end of the tube, and a steel backing plate is bonded to its bottom surface.

### 4.6 Quality Evaluation System

Using the acoustic‐electrical analogous TMM, we simplify the 3D spider‐like heteromorphic structure into four parallel 2D spider leg‐like theoretical models (Figure S2). We subsequently calculate the sound absorption performance of these abstracted 2D units using the TMM. Comparison curves validate the accuracy of this theoretical approach. CNN, with its local feature learning and multi‑task joint discrimination capabilities, automatically extracts spatial patterns from irregular structural images, enabling stable multi‑label performance determination. Therefore, the mapping between abstracted 2D images and sound absorption performance is established using a CNN. These leverage fitted curves from Figure S5a, with the CNN training database generated by parametrically deforming these curves. Randomly sampled validation sets achieve perfect accuracy [58, 59]. Based on this work, a three‐tier evaluation system for sound absorption bands is established, including low‐frequency (<2 kHz), mid‐frequency (2–6 kHz), and high‐frequency (>6 kHz) bands, with the labels rated as good, middle, or bad. Using geometric‐functional mapping, we implement quality evaluation of the underwater pressure‐resistant acoustic composite structure. The evaluation process is demonstrated in the Movie S3. This quality evaluation metamaterial also provides programmable and reconfigurable design ideas for underwater applications [60].

## Author Contributions

Conceptualization: Hongze Li, Zhenyu Li, and Jinshui Yang. Methodology: Hongze Li, Zhenyu Li, and Jianhao Wu. Investigation: Hongze Li, Zhenyu Li, Jianhao Wu, and Yidan Chen. Visualization: Hongze Li, and Yidan Chen. Supervision: Jinshui Yang, and Linzhi Wu. Writing – original Draft: Hongze Li, Zhenyu Li, and Jianhao Wu. Writing – review & Editing: Jinshui Yang, Linzhi Wu, Hong Hu, Penglin Gao, and Yegao Qu.

## Funding

The present work is supported by: The National Natural Science Foundation of China under Grant No. 12172098, U2141244. The State Key Laboratory of Mechanical System and Vibration under Grant No. MSV202404. The Shandong Provincial Natural Science Foundation under Grant No. ZR2024MA035.

## Conflicts of Interest

The authors declare no conflicts of interest.

## Supporting information




**Supporting File 1**: advs74834‐sup‐0001‐SuppMat.docx.

**Supporting File 2**: advs74834‐sup‐0002‐Movie S1.MP4.

**Supporting File 3**: advs74834‐sup‐0003‐Movie S2.MP4.

**Supporting File 4**: advs74834‐sup‐0004‐Movie S3.MP4.

## Data Availability

All data are available in the main text or the supplementary materials.
