# Supplementary material for: All‐in‐One Underwater Quality Evaluation Metamaterial With Mechanical Robustness, Sound Attenuation, and Diffuse Reflection
Source: Adv Sci (Weinh). 2026 Mar 14;13(30):e24261. doi: 10.1002/advs.202524261 (PMC13248777; doi:10.1002/advs.202524261)
Supplement: Supplementary file 1 — Supporting File 1: advs74834‐sup‐0001‐SuppMat.docx. [file ADVS-13-e24261-s004.docx]

**Supplementary Information for**

**All-in-one underwater quality evaluation metamaterial with mechanical robustness, sound attenuation, and diffuse reflection**

Hongze Li^1,2^, Zhenyu Li^3^, Jinshui Yang^1,2^*, Jianhao Wu^1,2^, Yidan Chen^2^, Linzhi Wu^1^*, Hong Hu^3^, Penglin Gao^4^, Yegao Qu^4^

1. College of Aerospace and Civil Engineering, Harbin Engineering University,

Harbin 150001, PR China.

2. Qingdao Innovation and Development Base, Harbin Engineering University,

Qingdao 266000, PR China.

3. School of Fashion and Textiles, The Hong Kong Polytechnic University,

Hung Hom, Hong Kong.

4. School of Mechanical Engineering, Shanghai Jiao Tong University,

Shanghai 200240, PR China.

*Corresponding author. Email: yangjinshui@hrbeu.edu.cn and wulinzhi@hrbeu.edu.cn

**Contents**

Supplementary Note 1: Transfer matrix method for optimization of acoustic-electrical analogies

3-5

Supplementary Note 2: Intelligent evaluation system and visualization interface setup

6-15

Supplementary Note 3: Impedance modulation and wave velocity reconstruction based on logic-gate screening 16-18

Supplementary Note 4: Mechanical robustness finite element computational methods

19-21

Supplementary Note 5: Preparation and assembly processes for components of underwater structures 22-25

Supplementary Note 6: Multi-objective performance of scaled-down structures

26-28

Supplementary Note 7: Promotion of metamaterial skin to underwater vehicle applications

29-30

Supplementary Note 8: Supplementary movies

31

References

32

**Supplementary Note 1: Transfer matrix method for optimization of acoustic-electrical analogies**

The transfer matrix method (TMM) exhibits poor adaptability to nonlinear complex structures. This limitation impedes the accurate prediction of structural sound absorption performance. Using the acoustic-electrical analogy method, we appropriately split the structure. This yields several simplified linear substructures compatible with the effective medium principle. Through the above approach, we derive the sound absorption performance for both the individual series-parallel substructures and the combined structure, as illustrated in Supplementary Figure 1.

Calculating the surface impedance using the TMM demands structural stratification (*1*). Each layer should exhibit maximal shape regularity and material homogeneity. Moreover, interfaces exhibiting distinct properties require delamination. Each layer height, as far as possible, needs to be <0.1λ_min_ to suppress the resonance peak shift. The layer transfer matrix **T*_i_*** and total transfer matrix **T** are given in Eqs. S1 and S2:

where $\text{k}_{\text{i}}$, $\text{d}_{\text{i}}$, and $\text{Z}_{\text{i}}$ denote the wave number, thickness, and equivalent impedance of each layer, respectively. The relationship between input/output external forces and vibration velocity gives:

where *F*_1_ and *u*_1_​ denote the normal force and velocity of the incident sound wave, while *F_n_*_+1_ and *u_n_*_+1_​ represent those at the rigid boundary. An ideal rigid backing suppresses vibration, implying zero velocity at the interface. Then, the structural overall surface impedance is calculated via the transfer matrix:

Next, we present the wave number and impedance solutions for individual layers. For viscoelastic layers such as polyurethane (PU) or rubber, the impact of loss factors on wave speed must be incorporated in the solution:

where $\text{ρ}_{\text{v}}$, $\text{E}_{\text{v}}$, $\text{v}_{\text{v}}$, and $\text{η}_{\text{v}}$ denote the density, Young's modulus, Poisson's ratio, and loss factor of viscoelastic materials, respectively. Using Eqs. S5-S6, we determine the equivalent wave number and impedance of the viscoelastic layer.

Structural layers containing gas domains necessitate domain equivalence, addressed through a corrective factor ($\text{ξ}$=*R_air_*/*R_layer_*) optimization model (*2*):

where $\text{E}_{\text{a}}$​ and $\text{ρ}_{\text{a}}$​ denote the equivalent modulus and density, respectively. Meanwhile, wave speed accuracy is enhanced by introducing Lamé constants to the equivalent model. The equivalent wave number and impedance of gas-containing structural layers are also derived from Eqs. S7-S8.

For composite material layers, acoustic-electrical analogy models the system as a parallel combination of materials. The equivalent impedance is derived as follows:

where $\text{Z}_{\text{mi}}$​ and $\text{S}_{\text{mi}}$​ represent the characteristic impedance and cross-sectional area of the parallel material element, respectively. Based on the above calculations, combined with the density of the equivalent medium in each layer, the equivalent wave speed can be deduced as:

Combining Eqs. S1-S4, the sound absorption coefficient is derived from the normal surface impedance. However, this method fails to accurately calculate nonlinear structures exhibiting steep impedance gradients. Conventional approaches employ effective medium theory for structural simplification with finite-element discretization, yet demand significant computational resources. Using acoustic-electrical analogy, we partition the structure topologically in a non-material viewpoint and reduce impedance gradients via series-parallel reduction (*3*). This method facilitates linear system construction as follows:

For geometrically straightforward configurations, the impedance transfer method applies effectively (*4*).

where $\text{Z}_{\text{para}}$ and $\text{Z}_{\text{seri}}$ denote the total parallel impedance and total series impedance, respectively. For simple systems, $\text{Z}_{\text{in, i}}$ is obtained by impedance inversion from bottom to top via the impedance transfer method.

Theoretical and numerical curves in Supplementary Figure 1 validate the structural partitioning method, showing consistent trends and reducing overall error by approximately 50%.





Supplementary Figure 1. Transfer matrix method optimized via electrical-acoustic analogy. a. Comparing analytical calculations and numerical simulations for PU end caps. b. i-ii are parallel units without end caps Z1 and Z2, iii-iv are modified parallel units with end caps Z1 and Z2. c. External framework impact analysis. d. Performance comparison- theoretical vs. simulated- for Z3-Z5 parallel units. e. Theoretical and simulated comparisons for equivalent combination structures 1 (through b. i-ii) and 2 (through b. iii-iv). f. Optimized theory enhances fit by nearly 50% over the initial TMM. The blue region represents sound absorption variations under structural numerical equivalence.

**Supplementary Note 2: Intelligent evaluation system and visualization interface setup**

This study establishes a sound attenuation evaluation system based on spider-inspired heterogeneous structures. The system incorporates a visualization interface that directly maps functional and geometric relationships. Unlike traditional methods employing axisymmetric rotation or two-dimensional contraction, spider-inspired structures do not permit dimensional simplification from 3D to 2D through the above strategy. Leveraging acoustic-electrical analogy, we apply parallel simplification to disassemble heterogeneous units within the centrosymmetric framework. Then, these discrete units are mapped to a simplified 2D configuration. The validity of the dimensional reduction approach is demonstrated by validating the structure's sound absorption properties during the simplification process (Supplementary Figure 2a).

Training (Data S1) and testing (Data S2) results show that the convolutional neural network (CNN) achieves prediction accuracy exceeding 90% after 20 training iterations. By the 50th iteration, the prediction error is nearly eliminated (Supplementary Figure 2b). Leveraging machine learning, we establish quality evaluation labels based on frequency-dependent acoustic performance. The classification criteria are defined as follows: (1) absorption peaks (α > 0.75) within 0-2 kHz; (2) absorption peaks (α > 0.8) in the 2-6 kHz range; (3) absorption peaks (α > 0.85) in the 6-10 kHz range. Meanwhile, labels classification is defined by requirement fulfillment: good for three criteria, middle for two, and bad for one.

For the 2D images in the randomly screened database, recognition yields corresponding evaluation labels (*5*). Furthermore, Data S3 implements the quality evaluation visualization interface. Representative cases of good, middle, and bad classifications are illustrated in Supplementary Figure 2c. This method characterizes multi-faceted performance of parallel structural components, reducing geometric complexity while increasing assessment specificity. It further facilitates performance evaluation and topological analysis of underwater structures.





Supplementary Figure 2. Dimension simplification and evaluation system testing. a. The parallel computational simplification approach and comparison of the sound absorption equivalent results. b. Validation of CNN learning efficacy. c. Visualization of intelligent evaluation system quality tag results.

Supplementary Data 1 CNN Training.

| **Data S1** CNN Training |
| --- |
| import tensorflow as tf  from time import *  import matplotlib  matplotlib.use('TkAgg')  import matplotlib.pyplot as plt  def data_load(data_dir, test_data_dir, img_height, img_width, batch_size):  train_ds = tf.keras.preprocessing.image_dataset_from_directory(  data_dir,  label_mode='categorical',  seed=123,  image_size=(img_height, img_width),  batch_size=batch_size)  val_ds = tf.keras.preprocessing.image_dataset_from_directory(  test_data_dir,  label_mode='categorical',  seed=123,  image_size=(img_height, img_width),  batch_size=batch_size)  class_names = train_ds.class_names  return train_ds, val_ds, class_names  def model_load(IMG_SHAPE=(436, 500, 3), class_num=3):  base_model = tf.keras.applications.MobileNetV2(input_shape=IMG_SHAPE, include_top =False, weights='imagenet')  base_model.trainable = False  model = tf.keras.models.Sequential([  tf.keras.layers.experimental.preprocessing.Rescaling(1./127.5, offset=-1, input_shape =IMG_SHAPE),  base_model,  tf.keras.layers.GlobalAveragePooling2D(),  tf.keras.layers.Dense(class_num, activation='softmax')  ])  model.summary()  model.compile(optimizer='adam', loss='categorical_crossentropy', metrics=['accuracy'])  return model  def show_loss_acc(history):  acc = history.history['accuracy']  val_acc = history.history['val_accuracy']  loss = history.history['loss']  val_loss = history.history['val_loss']  plt.figure(figsize=(8, 8))  plt.subplot(2, 1, 1)  plt.plot(acc, label='Training Accuracy')  plt.plot(val_acc, label='Validation Accuracy')  plt.legend(loc='lower right')  plt.ylabel('Accuracy')  plt.ylim([min(plt.ylim()), 1.02])  plt.title('Training and Validation Accuracy')  plt.subplot(2, 1, 2)  plt.plot(loss, label='Training Loss')  plt.plot(val_loss, label='Validation Loss')  plt.legend(loc='upper right')  plt.ylabel('Cross Entropy')  plt.title('Training and Validation Loss')  plt.xlabel('epoch')  plt.savefig('results/results_mobilenet-origin2.png', dpi=300)  plt.show()  def train(epochs):  begin_time = time()  train_ds, val_ds, class_names = data_load("./origindata/train", "./origindata/val", 436, 500, 3)  print(class_names)  model = model_load(class_num=len(class_names))  history = model.fit(train_ds, validation_data=val_ds, epochs=epochs)  model.save("models/mobilenet-origin2.h5")  end_time = time()  run_time = end_time - begin_time  print('time：', run_time, "s")  show_loss_acc(history)  if __name__ == '__main__':  train(epochs=50) |

Supplementary Data 2 CNN test.

| **Data S2** CNN test |
| --- |
| import tensorflow as tf  import matplotlib  matplotlib.use('TkAgg')  import matplotlib.pyplot as plt  import numpy as np  plt.rcParams['font.family'] = ['sans-serif']  plt.rcParams['font.sans-serif'] = ['SimHei']  def data_load(data_dir, test_data_dir, img_height, img_width, batch_size):  train_ds = tf.keras.preprocessing.image_dataset_from_directory(  data_dir,  label_mode='categorical',  seed=123,  image_size=(img_height, img_width),  batch_size=batch_size)  val_ds = tf.keras.preprocessing.image_dataset_from_directory(  test_data_dir,  label_mode='categorical',  seed=123,  image_size=(img_height, img_width),  batch_size=batch_size)  class_names = train_ds.class_names  return train_ds, val_ds, class_names  def test_mobilenet():  train_ds, test_ds, class_names = data_load("./origindata/train", "./origindata/test", 436, 500, 3)  model = tf.keras.models.load_model("models/mobilenet-origin2.h5")  loss, accuracy = model.evaluate(test_ds)  print('Mobilenet test accuracy :', accuracy)  test_real_labels = []  test_pre_labels = []  for test_batch_images, test_batch_labels in test_ds:  test_batch_labels = test_batch_labels.numpy()  test_batch_pres = model.predict(test_batch_images)  test_batch_labels_max = np.argmax(test_batch_labels, axis=1)  test_batch_pres_max = np.argmax(test_batch_pres, axis=1)  for i in test_batch_labels_max:  test_real_labels.append(i)  for i in test_batch_pres_max:  test_pre_labels.append(i)  print(test_real_labels)  print(test_pre_labels)  class_names_length = len(class_names)  heat_maps = np.zeros((class_names_length, class_names_length))  for test_real_label, test_pre_label in zip(test_real_labels, test_pre_labels):  heat_maps[test_real_label][test_pre_label] = heat_maps[test_real_label][test_pre_label] + 1  heat_maps_sum = np.sum(heat_maps, axis=1).reshape(-1, 1)  print(heat_maps_sum)  heat_maps_float = heat_maps / heat_maps_sum  show_heatmaps(title="heatmap", x_labels=class_names, y_labels=class_names, harvest= heat_maps_float, save_name ="results/heatmap_mobilenet-test2.png")  def show_heatmaps(title, x_labels, y_labels, harvest, save_name):  fig, ax = plt.subplots()  im = ax.imshow(harvest, cmap="OrRd")  ax.set_xticks(np.arange(len(y_labels)))  ax.set_yticks(np.arange(len(x_labels)))  ax.set_xticklabels(y_labels)  ax.set_yticklabels(x_labels)  for i in range(len(x_labels)):  for j in range(len(y_labels)):  ax.text(j, i, f"{harvest[i, j]:.4f}",  ha="center", va="center", color="black")  ax.set_xlabel("Predict label")  ax.set_ylabel("Actual label")  ax.set_title(title)  fig.tight_layout()  plt.colorbar(im)  plt.savefig(save_name, dpi=300)  plt.show()    if __name__ == '__main__':  test_mobilenet() |

Supplementary Data 3 Interface Settings.

| **Data S3** Interface Settings |
| --- |
| import tensorflow as tf  from PyQt5.QtGui import *  from PyQt5.QtCore import *  from PyQt5.QtWidgets import *  import sys  import cv2  from PIL import Image  import numpy as np  import shutil  import os  class MainWindow(QTabWidget):  def __init__(self):  super().__init__()  self.setWindowIcon(QIcon('images/logo.png'))  self.setWindowTitle('System')  self.model = tf.keras.models.load_model("models/mobilenet-origin2.h5")  self.to_predict_name = "images/good.png"  self.class_names = ['good', 'middle', 'bad']  self.resize(900, 700)  self.initUI()  def initUI(self):  main_widget = QWidget()  main_layout = QHBoxLayout()  font = QFont('Times New Roman', 15)  left_widget = QWidget()  left_layout = QVBoxLayout()  img_title = QLabel("Sample")  img_title.setFont(QFont('Times New Roman', 20))  img_title.setAlignment(Qt.AlignCenter)  self.img_label = QLabel()  img_init = cv2.imread(self.to_predict_name)  h, w, c = img_init.shape  scale = 436 / h  img_show = cv2.resize(img_init, (0, 0), fx=scale, fy=scale)  cv2.imwrite("images/show.png", img_show)  img_init = cv2.resize(img_init, (500, 436))  cv2.imwrite('images/target.png', img_init)  self.img_label.setPixmap(QPixmap("images/show.png"))  left_layout.addWidget(img_title)  left_layout.addWidget(self.img_label, 1, Qt.AlignCenter)  left_widget.setLayout(left_layout)  right_widget = QWidget()  right_layout = QVBoxLayout()  btn_change = QPushButton(" Upload ")  btn_change.clicked.connect(self.change_img)  btn_change.setFont(font)  btn_predict = QPushButton(" Identify ")  btn_predict.setFont(font)  btn_predict.clicked.connect(self.predict_img)  label_result = QLabel(' Result ')  self.result = QLabel("Waiting orders")  label_result.setFont(QFont('Times New Roman', 20))  self.result.setFont(QFont('Times New Roman', 28))  right_layout.addStretch()  right_layout.addWidget(label_result, 0, Qt.AlignCenter)  right_layout.addStretch()  right_layout.addWidget(self.result, 0, Qt.AlignCenter)  right_layout.addStretch()  right_layout.addStretch()  right_layout.addWidget(btn_change)  right_layout.addWidget(btn_predict)  right_layout.addStretch()  right_widget.setLayout(right_layout)  main_layout.addWidget(left_widget)  main_layout.addWidget(right_widget)  main_widget.setLayout(main_layout)  about_widget = QWidget()  about_layout = QVBoxLayout()  about_title = QLabel('Welcom')  about_title.setFont(QFont('Times New Roman', 24))  about_title.setAlignment(Qt.AlignCenter)  about_img = QLabel()  about_img.setPixmap(QPixmap('images/bj.jpg'))  about_img.setAlignment(Qt.AlignCenter)  label_super = QLabel("Made by：wuyiqiang")  label_super.setFont(QFont('Times New Roman', 16))  label_super.setAlignment(Qt.AlignRight)  about_layout.addWidget(about_title)  about_layout.addStretch()  about_layout.addWidget(about_img)  about_layout.addStretch()  about_layout.addWidget(label_super)  about_widget.setLayout(about_layout)  self.addTab(main_widget, 'Homepage')  self.addTab(about_widget, 'About')  self.setTabIcon(0, QIcon('images/homepage.png'))  self.setTabIcon(1, QIcon('images/about.png'))  def change_img(self):  openfile_name = QFileDialog.getOpenFileName(self, 'chose files', '', 'Image files(*.jpg *.png *jpeg)')  img_name = openfile_name[0]  if img_name == '':  pass  else:  target_image_name = "images/tmp_up." + img_name.split(".")[-1]  shutil.copy(img_name, target_image_name)  self.to_predict_name = target_image_name  img_init = cv2.imread(self.to_predict_name)  h, w, c = img_init.shape  scale = 500 / w  img_show = cv2.resize(img_init, (0, 0), fx=scale, fy=scale)  cv2.imwrite("images/show.png", img_show)  img_init = cv2.resize(img_init, (500, 436))  cv2.imwrite('images/target.png', img_init)  self.img_label.setPixmap(QPixmap("images/show.png"))  self.result.setText("Waiting for identifying")  def predict_img(self):  img = Image.open('images/target.png')  img = np.expand_dims(img, axis=0)  outputs = self.model.predict(img)  result_index = int(np.argmax(outputs))  result = self.class_names[result_index]  self.result.setText(result)  ftest_path = './images/'  outputpath = ftest_path + str(int(result_index)) + '.png'  img_path = os.path.join(outputpath)  labelpic = cv2.imread(img_path)  h, w, c = labelpic.shape  scale = 436 / h  img_show_result = cv2.resize(labelpic, (0, 0), fx=scale, fy=scale)  cv2.imwrite('images/show.png', img_show_result)  self.img_label.setPixmap(QPixmap("images/show.png"))  def closeEvent(self, event):  reply = QMessageBox.question(self,  'Quit',  "Do you want to close？",  QMessageBox.Yes | QMessageBox.No,  QMessageBox.No)  if reply == QMessageBox.Yes:  self.close()  event.accept()  else:  event.ignore()  if __name__ == "__main__":  app = QApplication(sys.argv)  x = MainWindow()  x.show()  sys.exit(app.exec_()) |

**Supplementary Note 3: Impedance modulation and wave velocity reconstruction based on logic-gate screening**

If the structural design exhibits an impedance mismatch with water, interfacial reflections can arise under sound excitation. In complex sound fields, such reflections may enhance backscattered echoes, thereby compromising acoustic stealth performance. Our design employs a scattering-based reconstruction method to broaden echo angles and suppress normal reflections in low-frequency bands where absorption is ineffective. When sound waves impinge on the array structure and are scattered omnidirectionally under conditions that correspond to the transition from Rayleigh scattering to Mie scattering, the process can be analogized to diffuse reflection from a rough surface. In such cases, the incident acoustic energy is widely dispersed, propagating in all directions. It thus achieves concurrent low-frequency diffuse reflection and broadband sound absorption.

Scattering reconstruction exploits impedance gradients and wave velocity anisotropy to distort incident wavefronts. This method simultaneously deflects the propagation paths of acoustic energy. Our design implements a logic-gate approach to regulate impedance by determining material consistency between the top plate and matrix layers. It further leverages the P-wave (longitudinal wave) to S-wave (transverse wave) velocity ratio to quantify wave-velocity anisotropy. Increasing anisotropy enhances wavefront distortion and ray deflection, causing reflected waves to deviate further from the normal direction. This effect widens the echo angle.

While maintaining the same effective vibrating area, we convert circular unit cells (conducive to experiments) to rectangular ones (facilitating array design), as shown in Supplementary Figure 3a. Curve comparisons can verify the accuracy of the equivalent method. By assessing wave-velocity anisotropy, the water-matching capability of surface and skin-equivalent structural impedance, and matrix hardness, we analyze how impedance modulation and wave velocity reconstruction influence diffuse reflection. Even in nominally isotropic materials, distinct P-wave and S-wave velocities arise due to their different bulk and shear moduli. For instance, the velocity ratio (*Cp*/*Cs*) typically approaches 1.8 in metallic materials. However, the high atomic density at metallic surfaces leads primarily to specular reflection of incident sound waves. Minimizing this reflection is critical for underwater acoustic stealth technology. Enhancing wave velocity anisotropy via composite structural design is the primary approach to trigger diffuse reflection. When the wave velocity ratio exceeds 2, matrix scattering typically transitions from P-wave to S-wave dominance. Diffuse reflections then originate primarily from subwavelength structural heterogeneities. In this design, the equivalent wave velocity ratio consistently exceeds 3.454 (reaching 6.612), except for hydrogel-matrix structures. This yields S-wave-dominated scattering, where structural shear effects primarily govern sound transmission. The critical scale ratio (heterogeneous feature size to S-wave wavelength) determines scattering regimes (*6*). Ratios near unity are avoided to promote uniform scattering. For the composite configurations in Fig. 5f and Supplementary Figures 3b-e, the calculated S-wave wavelengths range from 150 to 185 mm across operational frequency bands. Structural heterogeneities (approximately 20 mm characteristic size), which are slightly larger than one-tenth of the S-wave wavelength, enabling synergistic Rayleigh-resonance scattering. Notably, during the transition from Rayleigh to Mie scattering, the structure maintains omnidirectional (360°) scattering. It is characterized by a decrease in scattering uniformity, accompanied by an increase in scattering intensity. The above phenomenon arises from increased frequency or enlarged characteristic dimensions. Selecting the combination with low S-wave velocities (often associated with strong wave velocity anisotropy) can narrow the frequency range of Rayleigh scattering. This shift promotes enhanced scattering through vibrating mechanisms. Candidate combinations exhibiting these properties are: Shore90a PU matrix (Fig. 6f), Shore70a PU matrix, and Shore90a PU matrix with Shore70a PU top plate (Supplementary Figure 3e). Though the Shore70a PU-based structure exhibits superior acoustic performance. However, its low matrix hardness (compressive modulus: 10 MPa) compromises mechanical stability. Furthermore, the Shore90a PU-based structure exhibits similar impedance at the medium interface (*Rz*1) and skin-equivalent cover layers (*Rz*2). This confers significantly enhanced sound attenuation, diffuse reflection, and mechanical robustness compared to PU materials under analogous material properties.

Subsequently, we present the wave velocity distribution and sound attenuation effects for both the hydrogel plate combined with Shore 90a PU matrix and the hydrogel matrix alone. From Supplementary Figure 3f, we observe a peculiar phenomenon where the longitudinal vibration modes of the structure are shielded by the presence of the hydrogel material in the matrix, and a dramatic decrease in the S-wave velocity. Replacing the matrix material with PU, while retaining the hydrogel top plate, yields a negligible change in S-wave velocity. Material characterization reveals that the hydrogel's low shear modulus results in an extremely low S-wave velocity (2.15 m/s). Consequently, the shear modulus of the nylon-glass fiber (GF) frame substantially exceeds that of the hydrogel top plate, irrespective of the matrix material (hydrogel or PU). Under low-frequency excitation, the structural S-wave wavelength (>150 mm) significantly exceeds the hydrogel layer thickness (5 mm). Consequently, the wave is confined to oscillate within the hydrogel layer, and the substructure can be approximated as rigid. This results in S-wave velocity dominance by the hydrogel properties, maintaining a value near 6 m/s. Furthermore, the hydrogel's extremely low stiffness and high damping form an overdamped system with the embedded mass units. This eliminates structural resonance and suppresses longitudinal vibrations in the composite structure. Simultaneously, the inhomogeneous hydrogel layer (5 mm thick) is subwavelength (<1/10 S-wave wavelength), triggering Rayleigh scattering. Combined with its close acoustic impedance matching to water, this yields excellent sound attenuation and diffuse reflection characteristics. Although hydrogels exhibit lower stiffness, they remain viable as functional materials for near-water surface applications.


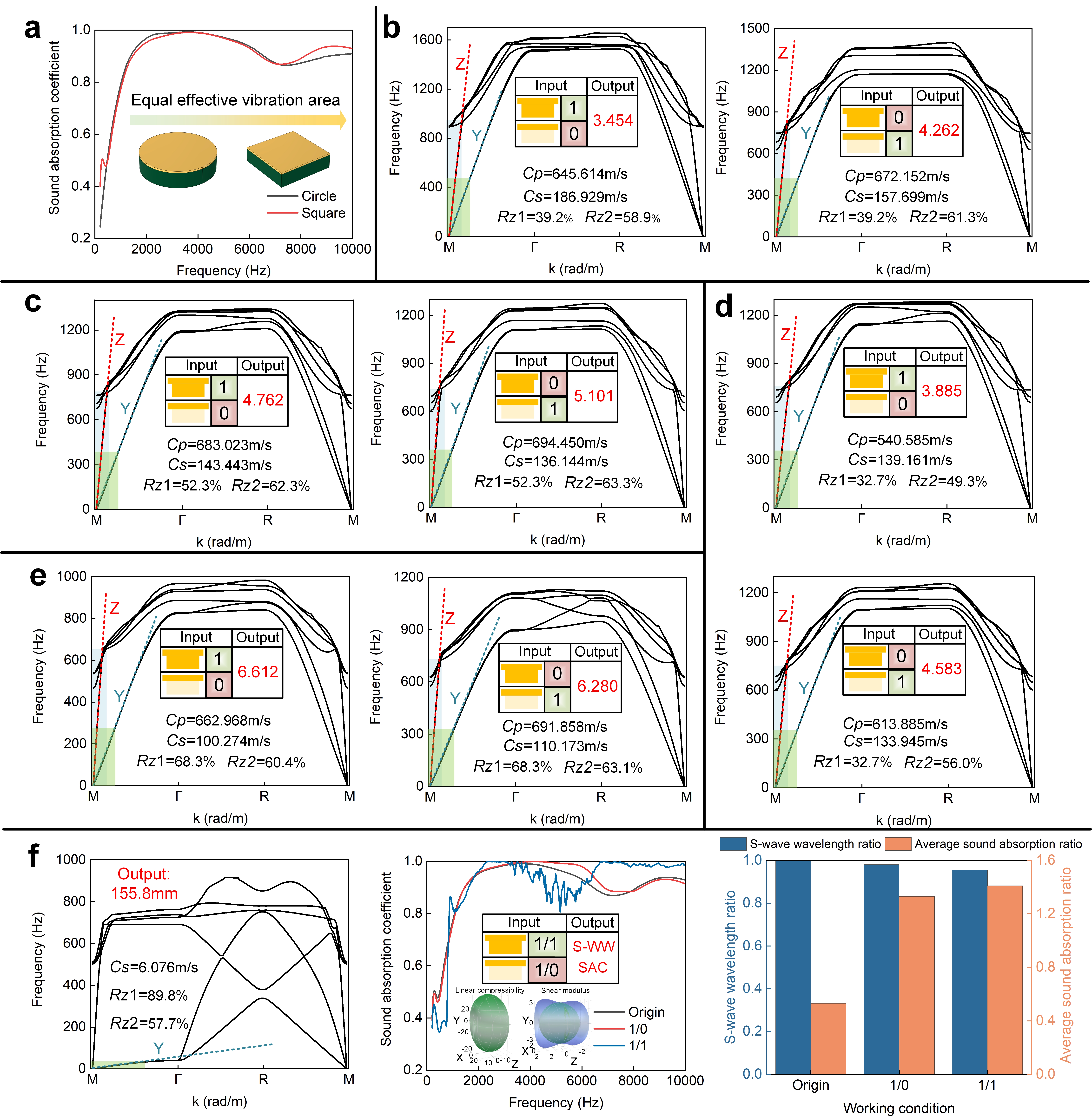


Supplementary Figure 3. Logic gate-based impedance modulation and wave velocity reconstruction. a. The cubic lattice exhibits equivalence under a constant effective vibrational area. b. Logic-gate screening enables impedance modulation and wave velocity reconstruction in the Shore 92A PU matrix. Among this, the orange full-coverage input defines a complete Shore 92A PU matrix. Conversely, the orange upper-panel input specifies a structure combining a Shore 92A PU upper panel with an additively manufactured Shore 90A PU interior. Results distinguish the wave velocity distribution by vibration pattern. The ratio of P-wave to S-wave velocity characterizes the level of anisotropy. Furthermore, *Rz* represents the corresponding impedance modulation results: *Rz*1 quantifies the water-to-surface layer impedance matching degree, and *Rz*2 quantifies the water-to-overlying skin impedance matching degree. Based on this, c-e denote the impedance and wave velocity modulation effects in three materials: 90A PU, glass microsphere-filled 90A PU, and 70A PU, respectively. f. S-wave wavelength governs the tunable sound attenuation in hydrogel-based composites.

Supplementary Note 4: Mechanical robustness finite element computational methods

The mechanical response of the structure under quasi-static compressive loading and uniform pressure is simulated using ABAQUS/Explicit finite element software. To mitigate challenges associated with difficult convergence stemming from extensive iterative processes, explicit methods are employed to enhance the model's convergence rate. The discrete rigid body model is used to construct the upper and lower platforms. The lower platform is completely fixed, and the displacements of the upper and lower platforms are suppressed except in the vertical direction (as shown in Supplementary Figure 4). Considering that the structure is completely fixed at the surroundings as well as at the bottom of the structure under hydrodynamic conditions, only the upper platform is replaced with a uniform pressure load when modeling the effect of uniform pressure on the structure. The mesh sensitivity analysis determined the average mesh size to be 0.5 mm, and the cells of the structure are created using an eight-node 3D reduced-integration solid cell (C3D8R). The type of contact in the simulation experiments is hard contact with a tangential friction coefficient of 0.15. To model the deformation and failure of composite structures, a vector user material subroutine (VUMAT) was implemented (*7*).

The modified 3D Hashin's failure criterion and Yeh delaminational failure criteria are used in the damage initiation model. In composite laminates, five damage modes can be summarized: fiber tensile fracture, fiber compression fracture, matrix tensile fracture, matrix compression fracture, and delamination failure. The corresponding five types of failure modes are defined as:

1. Fiber stretching ($\text{ε}_{11}\text{>0}$):
2. Fiber compression ($\text{ε}_{11}\text{≤0}$):
3. Matrix tension ($\text{ε}_{22}\text{+}\text{ε}_{33}\text{≥0}$):
4. Matrix compression ($\text{ε}_{22}\text{+}\text{ε}_{33}\text{<0}$):

$$\text{R}_{\text{mc}}^{2}\text{=}\left( \frac{\text{E}_{22}\text{ε}_{22}\text{+}\text{E}_{33}\text{ε}_{33}}{\text{G}_{12}\text{S}_{12}^{\text{ε}}} \right)^{2}\text{+}\left( \frac{\text{ε}_{22}\text{+}\text{ε}_{33}}{\text{Y}_{\text{C}}^{\text{ε}^{2}}} \right)\left[ \left( \frac{\text{E}_{22}\text{Y}_{\text{C}}^{\text{ε}}}{2\text{G}_{12}\text{S}_{12}^{\text{ε}}} \right)^{2}\text{-1} \right]\text{+}$$

5. Delamination failure ($\text{ε}_{33}\text{≥0}$):

where $\text{X}_{\text{T}}^{\text{ε}}$,$\text{ }\text{X}_{\text{C}}^{\text{ε}}$ are longitudinal tensile and compressive ultimate strain of composite laminates respectively, $\text{Y}_{\text{T}}^{\text{ε}}$,$\text{ }\text{Y}_{\text{C}}^{\text{ε}}$ are transverse tensile and compressive ultimate strain respectively, $\text{S}_{23}^{\text{ε}}$,$\text{ }\text{S}_{12}^{\text{ε}}$ and $\text{ }\text{S}_{13}^{\text{ε}}$ are in-plane and out-plane shear ultimate strain respectively, $\text{Z}_{\text{T}}^{\text{ε}}$ is tensile delamination ultimate strain. Failure factor $\text{R}_{\text{i}} \left( \text{i}\text{=}\text{ft}\text{, }\text{fc}\text{, }\text{mt}\text{, }\text{mc}\text{, }\text{ld} \right)$ represents the failure level. These limit strain components are defined as follows:

The damage variable $\text{d}_{\text{i}}$ is introduced into the damage evolution process to characterize the damage degree of composites. The definition domain of damage variable $\text{d}_{\text{i}}$ is [0,1]. If $\text{d}_{\text{i}}$= 0, there is no damage; when $\text{d}_{\text{i}}$=1, the composite material completely lost its mechanical properties. When damage occurs ($\text{d}_{\text{i}}$>0), the corresponding mechanical properties (elastic modulus) begin to deteriorate. The relationship $\text{d}_{\text{i}}$ with $\text{R}_{\text{i}}$ is shown in Equ. S25:

where *m* (*m*0) is the non-dimensional parameter to control the stiffness degradation rate of composite material. At time *t*, the damage variable as:

Based on damage variation, the stress-strain relationship of laminates can be expressed as:

where damage parameter $\text{ω}_{\text{i}}\text{=}\text{ }\text{(}\text{i}\text{=1,}\text{ }\text{⋯}\text{,}\text{ }\text{6)}$ is defined as:

According to the failure criterion, the unit stiffness will degenerate when a unit fails. Therefore, the maximum strain criterion is applied in the VUMAT subroutine to remove the distorted elements. Since the experiment has shown no signs of material failure. Consequently, in the simulation, we analyze only the extracted elastic-regime force-displacement curve.


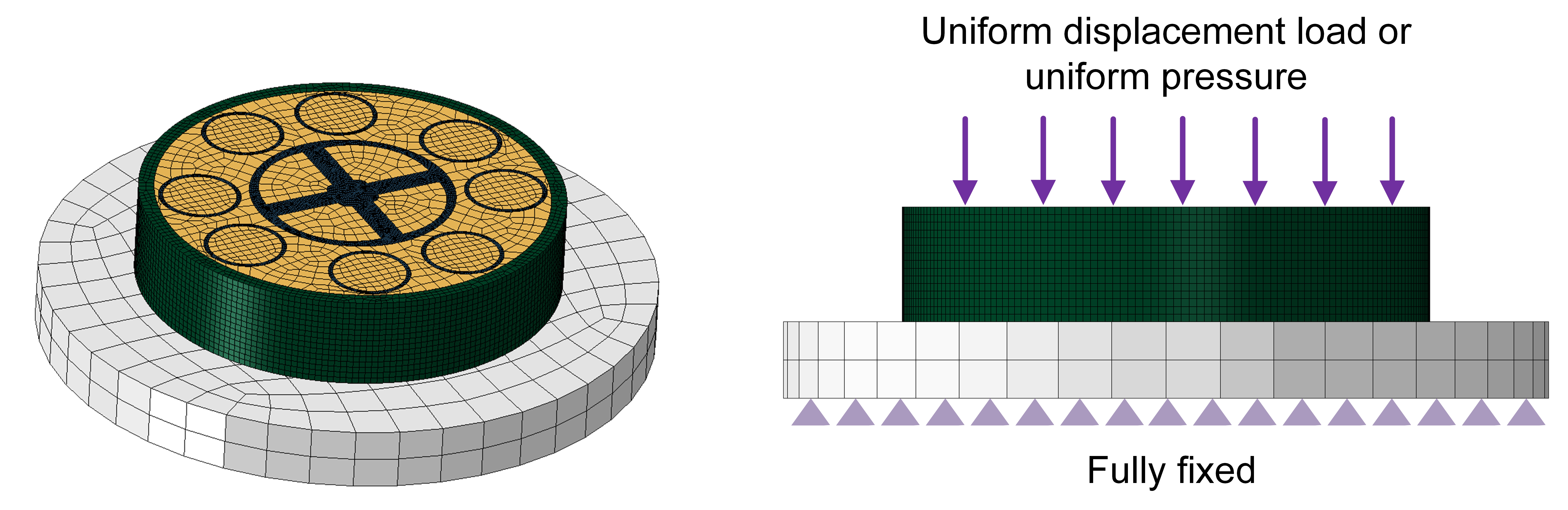


**Supplementary Figure 4. Numerical modeling setup approach for structural mechanics analysis.**

**Supplementary Note 5: Preparation and assembly processes for components of underwater structures**

The spider-inspired heteromorphic structure is presented through curve interpolation and rotational merging. This process is detailed in Supplementary Figure 5a. The structural complexity necessitates component-specific mold fabrication, imposing significant costs. This approach further impedes post-evaluation topology optimization for subsequent research on this structure. Furthermore, the infusion of viscoelastic materials is constrained by material preparation gradients in layered casting, which limits its manufacturing scale. Consequently, this process is reserved for the standard specimen fabrication. To address these limitations, we fabricate components via additive manufacturing using parameter-screened materials (supported by Figs. 3b-f, 6f, Supplementary Figure 3, and Supplementary Table 2).

Components are fabricated via stereolithography (SLA) and selective laser sintering (SLS), preparing GF reinforced nylon, PU, and synthetic rubber materials, respectively. Fabrication of the PU matrices, characterized by an irregular geometry and internal hollowed-out design, employs SLS. Uniquely, this process utilizes unsintered powder to support overhangs, thereby avoiding dedicated support structures and their challenging post-process removal. This inherent capability aligns perfectly with our structural fabrication objectives. Meanwhile, SLA is used to fabricate the nylon-GF outer frame and the spider-inspired heteromorphic structure, as these components lack complex internal skeletons. The printing procedure is shown in Supplementary Figure 5b.

Complementing additive manufacturing, we employ angle grinders, water jets, and engraving machines to cut carbon fiber plates, columns, and tubes fabricated from orthogonally laid prepregs. Meanwhile, additional steel blocks are prepared by lathe machining technology, and the complete structure is assembled by bonding. The structure is assembled as shown in Supplementary Figure 5c, and the constructed geometric parameters are shown in Supplementary Table 1. Furthermore, Supplementary Table 2 summarizes the linear elasticity constitutive parameters of distinct materials. For anisotropic carbon fibers, we define density, Young’s modulus, shear modulus, and Poisson’s ratio as: $\text{ρ}$=1570kg/m^3^, $\text{E}_{1}$=119Gpa, $\text{E}_{2}\text{,} \text{E}_{3}$=8.7Gpa, $\text{G}_{1}\text{,} \text{G}_{2}$=4Gpa, $\text{G}_{3}$=3Gpa, $\text{ν}_{1}\text{,} \text{ν}_{2}$=0.32, $\text{ν}_{3}$=0.38. Hydrogel parameters derive from wave velocities as: *Cp=*1546m/s, *Cs=*2.15m/s, $\text{ρ}$=1081kg/m^3^.


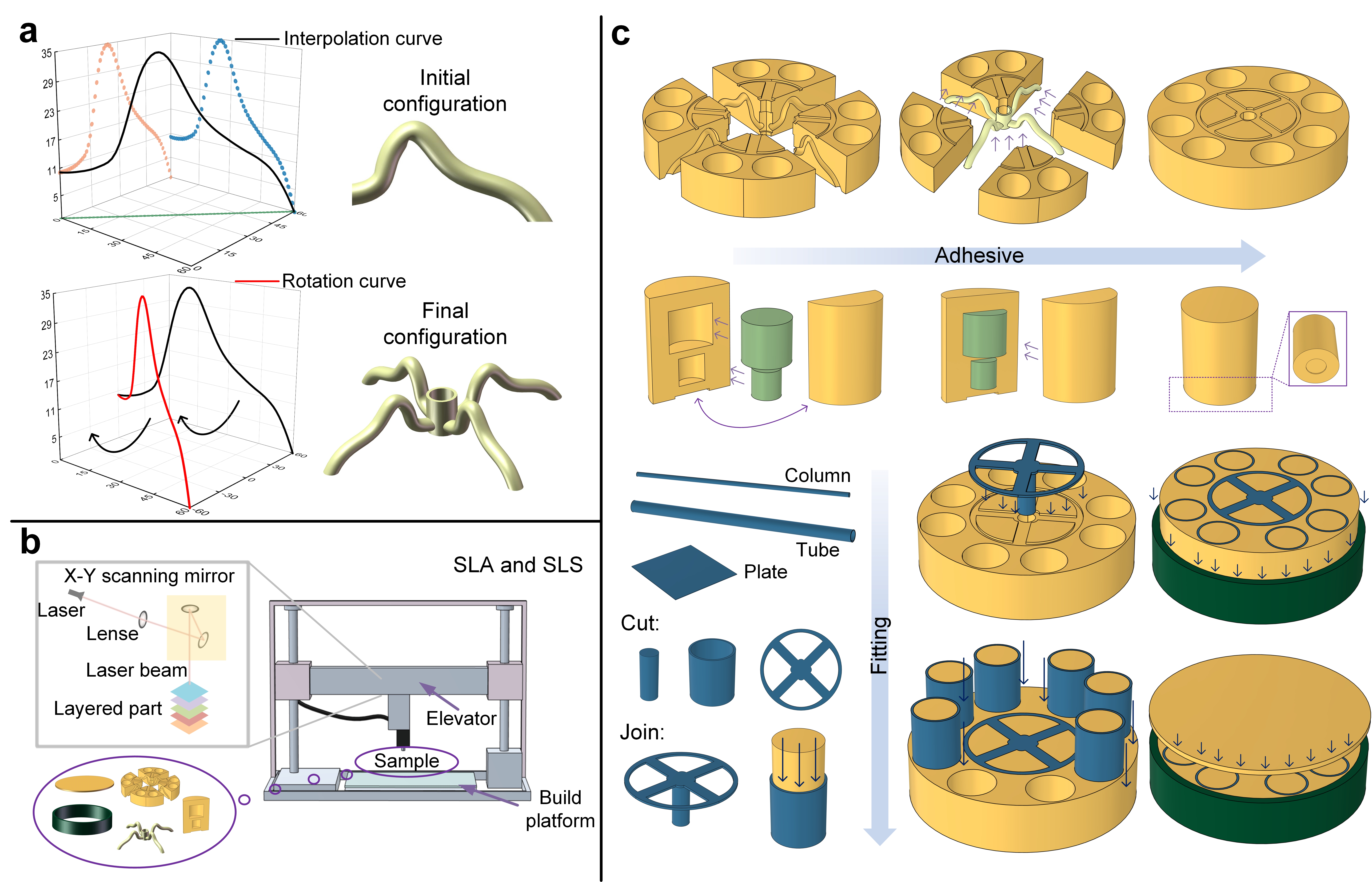


**Supplementary Figure 5. Underwater metamaterial skin’s design and fabrication. a.** Interpolation-enabled curve fitting in heteromorphic structures. **b.** Additive manufacturing of architected components. **c.** Structural preparation process.

Supplementary Table 1. Geometric parameters

| Parameters | a | b | c | d | e | f | g | h | l | m | t | b_1_ | b_2_ | b_3_ | b_4_ |
| --- | --- | --- | --- | --- | --- | --- | --- | --- | --- | --- | --- | --- | --- | --- | --- |
| Values  (mm) | 16 | 40 | 1 | 43 | 20 | 89.8 | 7.5 | 5 | 11.3 | 33 | 4 | 36 | 20 | 12 | 10 |
| Parameters | d_1_ | d_2_ | t_1_ | t_2_ | D | L | R | R_1_ | R_2_ | R_3_ | $\text{α}$ | $\text{β}$ | $\text{γ}$ | $\text{θ}$ |  |
| Values  (mm, °) | 18 | 12 | 15 | 20 | 45 | 74.3 | 96 | 5 | 38 | 50 | 35.3° | 54.7° | 19.5° | 19.7° |  |

Supplementary Table 2. Material parameters

|  | Young's modulus  (MPa) | Poisson's ratio | Density  (kg/m^3^) | Loss factor |
| --- | --- | --- | --- | --- |
| PU (Shore 70A) | 10 | 0.498 | 1100 | 0.8 |
| PU (Shore 90A) | 23.17 | 0.492 | 1100 | 0.8 |
| PU+Glass | 21.9 | 0.48 | 1100 | 0.8 |
| 3D printing PU | 17.38 | 0.496 | 1100 | 0.8 |
| PU (Shore 92A) | 43 | 0.472 | 1100 | 0.8 |
| Rubber | 1.054 | 0.489 | 1150 | 0.7 |
| Steel | 200000 | 0.33 | 7800 |  |
| Nylon-GF | 2810 | 0.32 | 1220 |  |

**Supplementary Note 6: Multi-objective performance of scaled-down structures**

This study addresses the challenges inherent in testing the broadband sound attenuation of large-scale structures with a 208 mm diameter impedance tube, as well as the load range limit resulting from their excessive surface area. By applying a scaling strategy, the structure is reduced to 118 mm, allowing it to be tested in a 120 mm impedance tube (Test Frequency Band: 0.5-10kHz) while simultaneously improving its stress tolerance. This approach enables simultaneous evaluation of acoustic and mechanical performance. Furthermore, the relative displacement curve and displacement distribution nephograms are employed to analyze the sound insulation mechanism. Meanwhile, mechanical robustness is examined through cyclic loading curves.

First, we have proportionally scaled down the structure using a factor of 1.754237. At this stage, maintaining integer dimensions for most structural components proves challenging. This constraint substantially increases fabrication difficulty and compromises precision. To simplify fabrication, the structural geometric parameters are rounded to integer values. This adjustment ensures that sound attenuation performance remains virtually unchanged. As a result, the components become easier to machine. When fabricating large-scale components, laboratory-scale vessels face size constraints that preclude monolithic casting of the matrix material. Employing a layered casting approach risks introducing unnecessary impedance mismatches caused by property variations between adjacent layers. To preserve manufacturing precision, additive manufacturing is ultimately adopted for the matrix configuration. However, the joints between segmented configurations are prone to generating unwanted scattering. This degrades the overall sound attenuation performance. The significant reduction in unit scale facilitates integrated casting during the fabrication process. This approach avoids material property inhomogeneity typically associated with layered casting. First, the PTFE mold is machined. Protrusions matching the volume of the structural cavity are incorporated at the mold base. This ensures that the cavity dimensions meet specifications. Second, the carbon fiber tubes are cut and positioned in the mold grooves. Then, the PU is poured at the marked locations. After curing, lathe machined steel blocks are bonded to the PU surface to prevent slippage during subsequent pouring steps. Next, the following components are placed at designated locations within the barrel shape mold: cast tubular components, cut carbon fiber plates and columns, additively manufactured spider-like heterogeneous structures, and nylon-GF frames. A PU layer is pre‑injected to secure their positioning. Finally, the specimen is fabricated via integrated PU casting and curing. The finished sample measures 118 mm in diameter and 30 mm in thickness, with a mass of only 446.67 g. The detailed casting process is shown in Supplementary Figure 6a.

Second, the sound attenuation performance of the samples is evaluated using impedance tube measurements. Numerical models are subsequently established based on the specimen configurations within the tube (*8*). The corresponding schematic diagrams are illustrated in the Supplementary Figures 6b and c. Additionally, a comparison of the numerical and experimental results for the sound absorption and insulation performance of the 118 mm structure is presented in Supplementary Figure 6d. The numerical model, configured based on the experimental environment, yields results that closely match the experimental data, confirming its reliability and accuracy. Meanwhile, the inverse relationship between geometric scaling and frequency is confirmed in Fig. 5e. Potential errors arise from fabrication-related parameter optimization and discrepancies in the scaling of water's added mass (*9*). Furthermore, the sound insulation mechanism is elucidated by correlating the relative displacement curve with the displacement nephograms at the peak and dip frequencies. Among these, point A represents the anti-resonance frequency, and point B represents the resonance frequency. The relative displacement curve provides a coordinated measure of the average out-of-plane displacement on the upper and lower surfaces of the structure. When the displacements on both sides are in the same direction and of comparable magnitude, the relative displacement approaches zero. Conversely, when they are in opposite directions, the relative displacement becomes pronounced. This offers an intuitive visualization of rigid‑body motion, contraction, and expansion within the structure. In the low-frequency stiffness-controlled segment, the relative displacement approaches zero. This indicates that the displacements on the upper and lower surfaces are in the same direction and of comparable magnitude, signifying that the structure undergoes rigid-body motion. At the anti‑resonance frequency, the relative displacement also approaches zero. This is manifested as a depression along the cross‑shaped opening on the upper surface and a corresponding protrusion on the lower surface, coinciding with the spider‑like heterostructure and cavity. The structure as a whole exhibits a contracted state, which prevents the relative displacement from reaching exactly zero. At this frequency, the structure exhibits minimal contraction and an exceptionally high equivalent stiffness. As a result, sound radiation is primarily reflected rather than transmitted through the structure, which accounts for the peak in sound insulation performance. At the resonance frequency, the relative displacement reaches a maximum. This is primarily attributed to intensified contraction similar to that at point A. Both soft and hard scatterers within the structure- such as the spider-like heterostructure and steel inserts- experience intense vibrational displacement. The structure readily alters its deformation state under acoustic excitation, resulting in low equivalent stiffness. Consequently, a significant portion of the incident sound radiation is transmitted, resulting in a minimum in sound insulation performance.

Finally, the equivalent compressive modulus of the structure is evaluated by five cyclic loading tests, as shown in Supplementary Figure 6e. The composite structure fabricated via the infusion process demonstrates excellent pressure resistance, with an equivalent compressive modulus of 72.75 MPa during the first loading cycle- approximately three times that of pure PU. During the 2nd to 5th loading cycles, the equivalent compressive modulus has consistently remained above 80 MPa. The structure has displayed instantaneous rebound, recovering approximately 90% of the applied strain, and has ultimately achieved nearly complete deformation recovery over time. This confirms the exceptional mechanical robustness of the proposed structure.


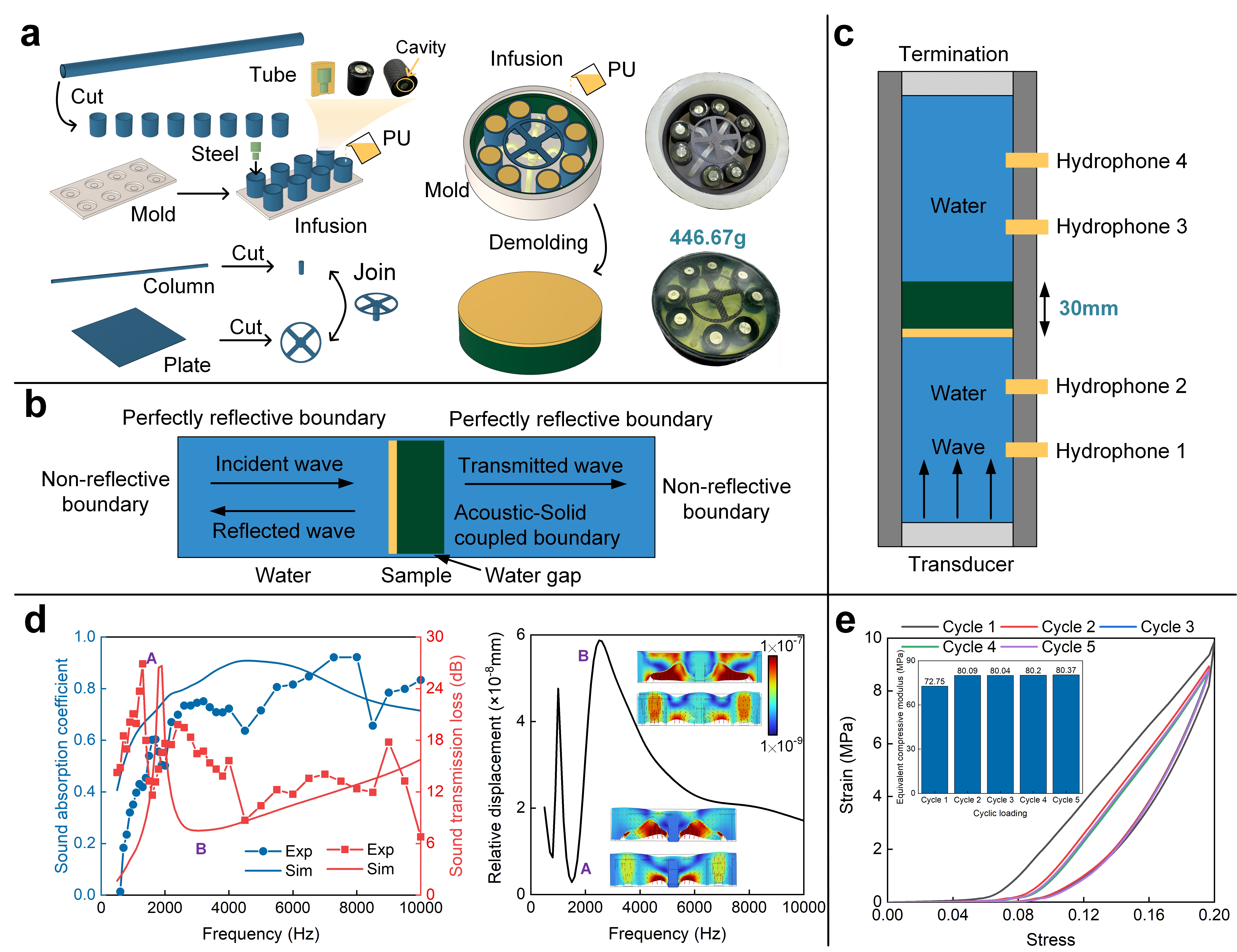


**Supplementary Figure 6. Multi-objective performance of the scaled model in acoustics and mechanics. a.** The structure fabrication process via infusion technology. **b.** Numerical model employed for simulating the sound insulation performance of the structure. **c**. Schematic of the impedance tube setup for the sound insulation experiment. **d**. Experimental and numerical comparison of sound absorption and insulation performance, with corresponding relative displacement curve and displacement field distributions. **e**. Cyclic loading curves of the scaled structure.

**Supplementary Note 7: Promotion of metamaterial skin to underwater vehicle applications**

This design combines sound attenuation, diffuse reflection, and hydrostatic pressure adaptation. Performance metrics for each parallel component enable evaluation, addressing actual engineering needs for multiscale design, performance predictability, and large-scale manufacturability. For underwater applications, the metamaterial skin can be applied to the vehicle hulls. It utilizes sound absorption and diffuse reflection to attenuate sound echoes and provide sound stealth. Additionally, to address varying water depths, particularly in deep-water environments (> 500 m), the mechanical skeleton enables efficient load-bearing and shape recovery. Under deep-water conditions, structural integrity is maintained while acoustic performance is minimally affected. When transitioning to shallower depths, acoustic performance recovers, enabling reversible deep-to-shallow cycling. For low-cost fabrication, large-scale mechanical skeletons incur lower manufacturing costs than small-scale honeycomb arrangements. Furthermore, high-volume production enables the use of large-scale molds for integrated PU casting. This approach eliminates material defects inherent in laboratory-scale layered casting and significantly reduces production costs. For installation, the structure can adopt either a square arrangement (preserving the effective vibration area) or a circular configuration with PU-filled gaps.





**Supplementary Figure 7. Potential engineering applications.**

Supplementary Note 8: Supplementary movies

Supplementary Movie 1.

Schematic representation of structure's sound attenuation and diffuse reflection performance

**Supplementary Movie 2.**

Schematic representation of the structure's hydraulic pressure adaptive performance

**Supplementary Movie 3.**

Quality evaluation system randomized testing process

**References**

1. H. Zou, L. Su, Y. Zhang, M. Zhang, W. Yu, X. Wang, X. Xia, H. Chen, X. Zhang, A. Zhao, A novel broadband underwater sound absorption metastructure with multi-oscillators. *Int. J. Mech. Sci.* **271**, 109137 (2024).
2. Z. Wang, Y. Huang, X. Zhang, L. Li, M. Chen, D. Fang, Broadband underwater sound absorbing structure with gradient cavity shaped polyurethane composite array supported by carbon fiber honeycomb. *J. Sound Vib.* **479**, 115375 (2020).
3. J. Feng, J. Qiao, Q. Xu, Y. Wu, G. Zhang, L. Li, Broadband Sound Absorption and High Damage Resistance in a Turtle Shell‐Inspired Multifunctional Lattice: Neural Network‐Driven Design and Optimization. *SMALL* **20**, 2403254 (2024).
4. X. Liu, C. Yu, F. Xin, Gradually perforated porous materials backed with Helmholtz resonant cavity for broadband low-frequency sound absorption. *Compos. Struct.* **263**, 113647 (2021).
5. J.-H. Wu, J.-S. Yang, X.-Y. Zhang, L.-L. Fu, S. Li, L.-Z. Wu, R. Schmidt, K.-U. Schröder, A neural network-based air-coupled ultrasonic damage detection method for composite honeycomb sandwich structure. *Mech. Syst. Sig. Process* **233**, 112789 (2025).
6. A. Roy, C. M. Kube, Multiple scattering of elastic waves in polycrystals. *J. Mech. Phys. Solids* **203**, 106237 (2025).
7. Z.-Y. Li, H.-Z. Li, J.-S. Yang, L. Ma, X.-T. Wang, Y.-Y. Gao, B.-G. Xu, J. Xiong, H. Hu, Multifunctional acoustic and mechanical metamaterials prepared from continuous CFRP composites. *Mater. Horiz.* **12**, 458-472 (2025).
8. C.-Q. Liang, M.-G. Wang, H.-B. Yang, J. Zhong, Z.-F. Zheng, Y. Wang, S.-S. Xu, J.-H. Wen, Pressure-resistant metastructures based on sliced-TPMS lattices for waterborne sound insulation. *Int. J. Mech. Sci.* **307**, 110851 (2025).
9. Q. Han, M. Li, Z. Wang, Evaluation on the applicability of similitude laws for scaled model design in underwater shaking table tests in the elastic stage. *Appl. Ocean Res.* **153**, 104252 (2024).
